# Regional inequality in infant RSV morbidity and mortality burden in the era of expanded RSV passive immunisation: a global projection modelling study

**DOI:** 10.1016/j.eclinm.2026.104219

**Published:** 2026-09-23

**Authors:** Shaolong Ren, Bingbing Cong, Jing Zou, Ling Guo, Harish Nair, Erin Sparrow, Daniel R. Feikin, You Li

**Affiliations:** a Department of Epidemiology, National Vaccine Innovation Platform, School of Public Health, Nanjing Medical University, Nanjing, China; b Key Laboratory of Public Health Safety and Emergency Prevention and Control Technology of Higher Education Institutions in Jiangsu Province, Nanjing Medical University, Nanjing, China; c Centre for Global Health, Usher Institute, University of Edinburgh, Edinburgh, UK; d Department of Immunizations, Vaccines, and Biologicals, World Health Organization, Geneva, Switzerland; e Changzhou Third People's Hospital, Changzhou Medical Centre, Nanjing Medical University, Changzhou, China

**Keywords:** Respiratory syncytial virus, Disease burden, Health equality, Passive immunisation, Nirsevimab, RSVpreF, Infants

## Abstract

**Background:**

Respiratory syncytial virus (RSV) is a major cause of acute lower respiratory infection (ALRI) in infants, with low- and middle-income countries (LMICs) bearing a disproportionately high burden. While novel RSV prophylactic products including long-acting monoclonal antibodies and a maternal vaccine are being rolled out for protecting the general infant population in high-income countries, implementation in LMICs is only expected by 2028, potentially exacerbating health inequities. We aimed to assess regional inequality in infant RSV disease burden with and without the implementation of these interventions.

**Methods:**

We re-analysed the previous global RSV disease burden dataset in infants by WHO regions and World Bank Income Classification for the year of 2019 as a baseline, using generalised linear mixed-effects models. We developed a static deterministic model that explicitly accounted for the varied risks for RSV admission by birth month and chronological age in month and projected the annual RSV-associated ALRI hospital admissions and RSV-attributable deaths under two scenarios: a no-implementation scenario that assumed the same hospital admission and mortality rates to the 2019 baseline, and a June-2025 product use scenario that was based on country-level implementation status of novel RSV prophylactic products as of June 2025; the two scenarios were then compared to estimate the impact of the current implementation of novel prophylactic products. We further calculated the ratio of RSV-attributable deaths between the highest and lowest burden quintiles (Q5-Q1 ratio) to assess inequality under the two scenarios.

**Findings:**

At baseline, the African Region had the highest incidence of RSV-associated ALRI (117.4 per 1000 person-years, 95% uncertainty range, UR: 60.0–229.8) and highest RSV-attributable mortality rate (0.97 per 1000 person-years, 95% UR: 0.76–1.31) while having the lowest RSV-associated ALRI hospital admission rate (12.6 per 1000 person-years; 95% UR: 7.5–21.3); the Eastern Mediterranean Region had the highest hospital admission rate (29.9 per 1000 person-years, 95% UR: 14.2–63.2). The regional variations in incidence, hospital admission, and mortality rates persisted when further stratifying the above estimates by country income level. As of June 2025, nirsevimab and maternal RSVpreF had been licensed in 54 and 64 countries, respectively, with 16 and 12 countries introducing them into their national immunisation programmes. A total of 4,641,000 doses of nirsevimab and 1,955,000 doses of RSVPreF were estimated to be administered annually under the June-2025 product use scenario globally. Administration of both products was projected to jointly avert 91,000 (95% UR: 75,000–118,000) RSV-associated ALRI hospital admissions and 300 (95% UR: 210–470) RSV-attributable deaths in infants annually, corresponding to 3.7% (95% UR: 2.7–4.9) and 0.4% (95% UR: 0.3–0.7) of the global admissions and deaths, respectively. Approximately 90% of averted hospital admissions and over 70% of averted deaths in infants occurred in high-income countries (81,000 [95% UR: 66,000–108,000] and 220 [95% UR: 140–390], respectively), corresponding to reductions of 33.2% (95% UR 31.0–35.9) and 29.1% (95% UR 22.6–35.2); by contrast, the relative reduction of admissions and deaths in middle-income countries was less than 5%, and no reduction was expected in low-income countries. Globally, substantial inequality was observed in the RSV-attributable overall mortality burden under the no-implementation scenario, with the Q5-Q1 ratio of RSV-attributable deaths estimated at 18.3 (95% UR: 11.7–27.5). The June-2025 product use scenario was associated with a possible slight increase in global inequality, with the Q5-Q1 ratio rising to 20.8 (95% UR: 13.0–31.7).

**Interpretation:**

The global RSV burden in 2019 varied substantially by region and income level, with the African Region exhibiting the highest incidence and mortality but the lowest hospitalisation rates. The current implementation of novel RSV prophylactic products, characterised by widespread licensing and use in high-income countries and lack of availability in LMICs, particularly the African Region, is projected to yield only modest reductions in the global infant RSV disease burden. Critically, inequitable access to these products may exacerbate existing inequalities in RSV mortality burden. These findings underscore the urgent need for targeted strategies to accelerate the introduction and scale-up of infant RSV immunisation programmes in LMICs.

**Funding:**

World Health Organization and National Natural Science Foundation of China (82473692).

**Research in context**

**Evidence before this study**

Respiratory syncytial virus (RSV) is a leading cause of acute lower respiratory infection (ALRI) and mortality in infants worldwide. We searched PubMed for studies published up to September 1, 2025, using the terms “respiratory syncytial virus”, “RSV”, “infants”, “burden”, “mortality”, “hospitali∗”, “maternal vaccine”, “monoclonal antibod∗”, “nirsevimab”, “RSVPreF” and “impact”. Previous studies have shown that the global disease burden of RSV is unequally distributed, with over 95% of RSV-associated ALRI episodes and more than 97% of RSV-attributable deaths occurring in low- and middle-income countries (LMICs). Following the approval of novel RSV immunisation products, implementation of these products has progressed rapidly in several high-income countries. Nirsevimab uptake exceeded 80% in Spain and Luxembourg; in the USA, the uptake of nirsevimab and RSVpreF (in pregnant women, same hereafter) has reached 51% and 31%, respectively; the uptake of RSVpreF in the UK was 55%. In contrast, implementation remains limited in low-income settings, which face the highest RSV morbidity and mortality burden. Although several modelling studies assessed the expected impact of nirsevimab and RSVpreF on RSV disease burden in infants, these analyses primarily focused on selected high-income or single countries, without considering regional disparities across the globe.

**Added value of this study**

To our knowledge, this is the first global study to assess regional inequality in infant RSV disease burden and their exacerbation amid the rollout of novel RSV passive immunisation. By leveraging comprehensive global RSV epidemiology data including disease burden, seasonality, hospitalisation risk by birth month and age in month and data on country-level implementation status and uptake of novel RSV prophylactic products, we developed a static deterministic model to analyse the impact of RSV prophylactic products on RSV-associated ALRI hospital admissions and RSV-attributable deaths across WHO and income regions. In the 2019 baseline, the African Region exhibited the highest RSV-associated ALRI incidence and RSV-attributable mortality, but the lowest RSV-associated ALRI hospitalisation rates; Eastern Mediterranean Region had the highest hospital admission rate. Based on the implementation status of novel RSV prophylactic products as of June 2025, our projections highlight that while high-income countries are likely to experience substantial reductions in infant RSV-associated ALRI hospitalisations (33.2%) and RSV-attributable deaths (29.1%), the public health benefits in LMICs, particularly those in the African Region, remain minimal under the current status of implementation. The inequitable access to these interventions exacerbates the pre-existing cross-country mortality inequalities, with the ratio of infant RSV-attributable mortality between the highest- and lowest-burden population quintiles increasing from 18.3 to 20.8.

**Implications of all the available evidence**

The accumulated evidence indicates that effective interventions for reducing severe infant RSV disease, specifically long-acting monoclonal antibodies and maternal vaccines, are now available and are being deployed increasingly in high-income settings. However, without equitable and accelerated introduction in LMICs, which bear the highest RSV mortality burden, current implementation will fail to address global inequities in child health. While the Gavi board has approved an RSV maternal immunisation programme in July 2025, this is not expected to result in vaccine introduction until 2028, and is expected to first roll-out in only a few Gavi eligible countries. Policymakers, international organisations, and manufacturers should continue to prioritise sustainable financing, supply allocation, and regulatory support to enable rapid and widespread adoption of RSV prophylactics in resource-limited settings.

## Introduction

Respiratory syncytial virus (RSV) is a leading cause of acute lower respiratory infection (ALRI) in infants and young children.^1^ In 2019, there were 33.0 million RSV-associated ALRI episodes, 3.6 million hospitalisations and more than 100,000 deaths in children younger than 5 years worldwide; notably, over 95% RSV-associated ALRI episodes and over 97% RSV-attributable deaths occurred in low- and middle-income countries (LMICs).^2^

Recent advancements in the development of RSV prophylactic products targeting infants, including long-acting monoclonal antibodies nirsevimab (licensed in 2022) and clesrovimab (licensed in 2025), as well as the maternal vaccine RSVpreF (licensed in 2023), offer promising solutions to reduce RSV morbidity and mortality. The World Health Organization's (WHO) Strategic Advisory Group of Experts on Immunization recommends all countries introduce maternal vaccine and long-acting monoclonal antibodies for the prevention of severe RSV disease in infants.^3^ Both nirsevimab and RSVpreF have demonstrated high real-world effectiveness against RSV hospitalisation and severe outcomes.^4^^,^^5^ According to a meta-analysis across four countries, nirsevimab was 83% effective against RSV hospitalisation during the first RSV season of the infants^4^; a test-negative study in Argentina reported 71% effectiveness of RSVpreF against RSV hospitalisation up to 6 months of life.^5^

However, these products were approved and used mainly in high-income countries (HICs); by comparison, access was limited in upper-middle-income countries and there was no access in lower-middle-income or low-income countries.^6^ This disparity raises concerns that delayed introduction of RSV prophylactics in these LMICs may widen regional inequalities in RSV disease burden; HICs start to witness declining incidence rates of RSV severe diseases while LMICs continue to bear the highest burden. Therefore, it is critical to understand the extent to which the imbalanced implementation of these novel RSV prophylactic products may further exacerbate regional disparities in RSV morbidity and mortality to inform policy.

In this study, we first quantified inequalities in infant RSV morbidity and mortality burden across WHO regions (African Region, Eastern Mediterranean Region, European Region, Region of the Americas, South-East Asia Region, and Western Pacific Region) and country income levels according to the World Bank's 2019 classification (low-, lower-middle-, upper-middle-, and high-income countries) within those regions (Appendix pp 3–8) using 2019 pre-implementation data. We then projected the annual RSV hospitalisation and mortality burden under two scenarios: a June-2025 product use scenario based on implementation status as of June 2025 and a scenario with no implementation, to understand how differential implementation status of RSV prophylactic products may reshape global inequities in RSV disease burden in infants.

## Methods

### Data collection and processing

#### RSV disease burden

We utilised the data on RSV disease burden from our previously published global RSV morbidity and mortality burden study in young children.^2^ The data were collected through the Respiratory Syncytial Virus Global Epidemiology Network (RSV GEN) in 2019. In this study, we used RSV disease burden estimates derived from these data as the baseline to represent RSV disease burden prior to the introduction of novel prophylactic products. The following four outcomes were included: RSV-associated acute lower respiratory infection (ALRI) in the community, RSV-associated ALRI hospitalisation, RSV-associated ALRI in-hospital deaths, and RSV-attributable overall deaths. For the four outcomes, we included the data in infants aged 0–<12 months in this study (details in Appendix pp 12–27).

For the two outcomes, RSV-associated ALRI in the community and RSV-associated ALRI hospitalisation, when data specific to the infant age group 0–<12 months were not available in the included studies, we utilised data in children aged 0–<24 months, 0–<36 months or 0–<60 months where available and conducted multiple imputation to estimate the incidence rate for 0–<12 months. Briefly, the imputation method, as used in the previous studies on disease burden of RSV and influenza virus in children,^2^^,^^7^ was based on the assumption that the logarithm incidence rate ratio (IRR) between two specific age groups (e.g., 0–<12 months and 0–<24 months) followed the same statistical distribution per outcome. For each original incidence rate estimate in 0–<24 months, 0–<36 months or 0–<60 months, we generated 1000 imputation samples of incidence rate for 0–<12 months by applying the corresponding IRR estimates (1000 samples drawn from log-normal distribution of meta-estimated IRR based on data sources with available data, details in Appendix p9).

#### Country-level implementation status of nirsevimab and RSVpreF

Information on the country-level licensing and implementation status of the long-acting monoclonal antibody nirsevimab and the maternal RSV vaccine RSVpreF as of June 2025 was collected through web searches using keywords including “nirsevimab”, “RSVpreF”, “monoclonal antibod∗”, “maternal vaccine”, “RSV”, “licens∗”, and “approv∗”, or their relevant local-language equivalents including local brand names, each combined with country names. The search results were cross-checked against official national regulatory and health authority websites, published literature, and further validated and supplemented with the latest internal WHO data. We classified the implementation status of nirsevimab and RSVpreF into three mutually exclusive groups: licensed and included in the National Immunisation Program (NIP), licensed but not included in NIP, and unlicensed. We developed an interactive visualisation tool to present the global implementation status of nirsevimab and RSVPreF.

#### Country-level uptake of nirsevimab and RSVpreF

Country-level uptake data of nirsevimab and RSVpreF in Spain, US, France, and Luxembourg were primarily identified from a recent systematic review and meta-analysis.^8^ For Chile, Italy, Ireland, UK, and Argentina, uptake data were obtained from official reports or published studies.^9^, ^10^, ^11^, ^12^, ^13^ For countries without available uptake data, uptake was assumed according to the implementation status of nirsevimab and RSVpreF, while also considering affordability if the product was available only in the private market; moreover, for RSVpreF, we referred to the real-world uptake of the maternal tetanus, diphtheria, and acellular pertussis (Tdap) vaccine, which has an overlapping administration window with RSVpreF. Specifically, when nirsevimab was included in the NIP as the primary product, 80% uptake was assumed. When RSVpreF was the primary NIP product, uptake was set at 60% or at Tdap coverage, whichever was lower. For countries where either product was only available in the private market, country-income-dependent uptake was applied; for the same country income level, RSVpreF uptake assumed to exceed that of nirsevimab, reflecting the anticipated price differences in the private market. Sensitivity analysis exploring lower and higher uptake assumptions were undertaken to assess the robustness of our projections. More detailed description of the assumed uptake can be found in Appendix pp 30–34).

#### Month-by-month age distribution of RSV hospitalisations

We utilised estimates on the distribution by month of age of RSV hospitalisation among infants from our published study,^14^ which applied a hierarchical Bayesian model to synthesise data from various sources. The estimates represented the proportions of RSV hospitalisation in each month of age during the first year of life. In the original study, the estimates were available by income level and by latitudinal groups; for this analysis, we re-ran the Bayesian model to further yield the estimates by WHO region and income level (Appendix pp 35–37).

#### RSV seasonality

For countries where nirsevimab or RSVpreF was licensed, we identified and included data on month-by-month RSV activity (excluding the years of 2020–2022 due to the transient impact of the COVID-19 pandemic on RSV) through a stepwise approach. As the first step, we searched relevant national surveillance data from official sources (e.g., the national centre for disease prevention and control). If no relevant data were identified, a literature search was conducted in PubMed using terms “seasonality,” “respiratory syncytial virus,” (or its equivalent forms) and “epidemi∗ characteristics,” combined with country names for identifying relevant published studies. In the absence of data from surveillance and published studies, RSV seasonality in a country was assumed to be the same as countries within the same hemisphere (i.e., northern or southern) and latitudinal region (tropical: 0°–23.5°, subtropical: 23.5°–35°, temperate: ≥35°) given that RSV seasonality was previously shown to be highly associated with latitudes (details in Appendix pp 38–46).^15^

### Data analysis

We first estimated the baseline RSV disease burden by WHO region and income level in 2019 and then projected the annual RSV hospitalisation and mortality burden under two scenarios: the no-implementation scenario where no countries introduced nirsevimab or RSVPreF and the June-2025 product use scenario where countries maintained their status of implementation as of June 2025 (Fig. 1).

**Fig. 1:**
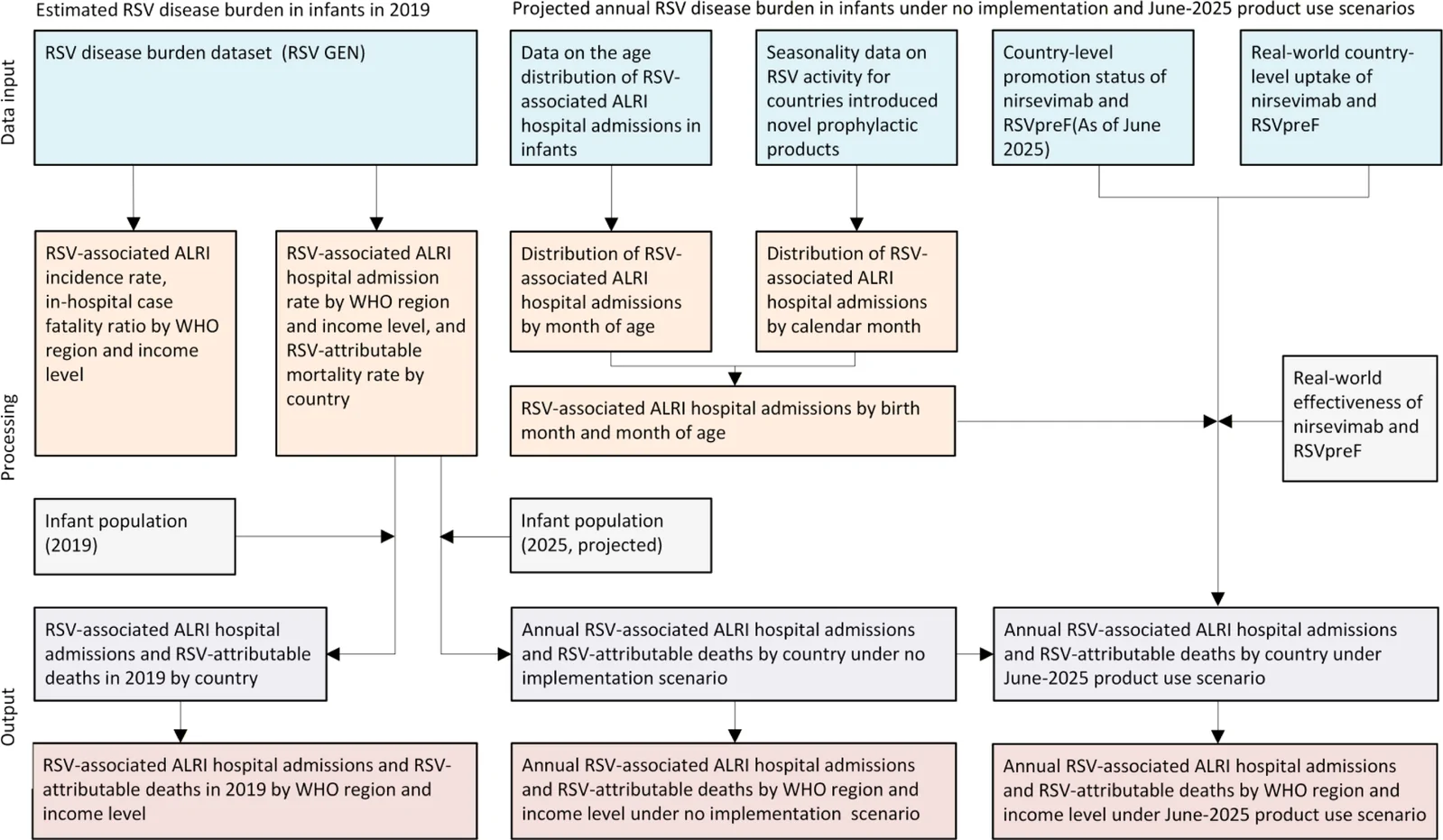
Schematic figure presenting the workflow of estimating and projecting regional RSV morbidity and mortality burden in infants. RSV = respiratory syncytial virus. ALRI = acute lower respiratory infection.

#### Estimating RSV disease burden in 2019

We applied the same generalised linear mixed-effects model (GLMM) as previously^2^ for estimating RSV-associated ALRI incidence rate, hospital admission rate, and in-hospital case fatality ratio (hCFR) by WHO region and income level (detailed methods can be found in Appendix pp 10–11); for overall mortality burden, we used the previously developed meta-regression model to estimate country-level RSV-attributable all-cause mortality estimates and aggregated the estimates by WHO region and income level.^2^ We calculated the number of RSV-associated ALRI episodes, hospital admissions, in-hospital deaths, and overall attributable deaths by applying the population data in 2019. We further assessed the regional disparities by visualising the relationship between RSV-associated ALRI incidence rate with RSV-associated admission rate and with RSV-attributable mortality rate across different WHO regions and income levels.

#### Projecting RSV-associated hospitalisation and mortality burden

We first projected overall annual RSV-associated ALRI hospital admissions and RSV-attributable deaths among infants under the no-implementation scenario, by applying the estimated RSV-associated hospital admission rate and RSV-attributable mortality rate in 2019 to the country-level infant population for 2025 (representing the June-2025 population size), assuming that RSV morbidity and mortality rates in 2025 were comparable to pre-pandemic levels without the implementation of the novel prophylactic products; this assumption was supported by relevant epidemiological studies^16^, ^17^, ^18^ and recent surveillance data from several countries during the 2024-25 season,^19^, ^20^, ^21^ which showed that the intensity of RSV activity had returned to the pre-pandemic level. Subsequently, we applied a static deterministic model to estimate the annual country-level RSV-associated ALRI hospital admissions and RSV-attributable deaths under the June-2025 product use scenario. The model explicitly accounted for the varied hospitalisation risks by age in months and birth month following our previously developed Bayesian hierarchical model (Appendix pp 35–37).^14^ For nirsevimab, we considered a seasonal immunisation strategy for all countries that licensed nirsevimab as of June 2025 with a birth dose approach for those born during the local RSV season and a catch-up dose approach for those aged <7 months at the season onset (to ensure that all infants up to the age of 12 months could be protected). For RSVPreF, we assumed that all countries that licensed RSVPreF as of June 2025 used a year-round approach except five countries (Austria, Belgium, the United States, Argentina, and Uruguay) that recommended a seasonal approach. In countries where both products were available, maternal vaccination and nirsevimab were assumed to be mutually exclusive, with infants protected by maternal vaccination excluded from nirsevimab eligibility to avoid double-counting of protection. In the main analysis, we assumed an effectiveness of 83% for nirsevimab against RSV-associated ALRI hospital admission and 81% against RSV-attributable death, and an effectiveness of 71% and 77%, respectively, for RSVPreF, informed by real-world estimates of effectiveness against severe RSV outcomes.^4^^,^^5^ We assumed constant effectiveness over the six-month protection period and did not model waning of protection, given the limited evidence available on the time-varying effectiveness of these products. Effectiveness was assumed to be the same for birth-dose and catch-up-dose recipients, consistent with recent real-world evidence showing comparable protection against RSV hospitalisation following a single nirsevimab dose across these groups.^22^ More detailed methodology can be found in Appendix (pp 47–52).

#### Assessing regional inequality in mortality burden

We assessed inequality in RSV mortality burden by ranking the population according to country-specific RSV-attributable mortality rates and dividing the population into quintiles according to these rankings. For the global analysis, quintiles were defined using the ranking of all countries globally, whereas for analyses within WHO regions, countries were ranked and grouped into quintiles separately within each region. We then calculated the ratio of RSV-attributable deaths between the highest and lowest quintiles (i.e., Q5-Q1 ratio); a Q5-Q1 ratio of 1 indicates perfect equality and higher Q5-Q1 ratio indicates greater inequality. We compared the Q5-Q1 ratios between the no-implementation scenario and the June-2025 product use scenario to assess the potential change in global inequality associated with the implementation of RSV prophylactic products.

#### Uncertainty and sensitivity analysis

To propagate uncertainty in the projected RSV-associated hospitalisation and mortality burdens, we generated 1000 simulation draws for each input parameter. For hospitalisation rates, draws were sampled from the log-normal distributions of the estimates derived from the GLMM meta-analysis. For mortality rates, the proportions of RSV-attributable deaths among all-cause deaths were sampled from logit-normal distributions based on predictions from the meta-regression model. For the proportions of RSV-associated ALRI hospital admissions by age in months and by birth month, draws were sampled from their respective posterior distributions. The effectiveness and uptake of the immunoprophylactic products were treated as fixed values. Draws from the different input parameters were combined one-to-one to generate 1000 estimates of the projected outcomes. The 95% uncertainty ranges (URs) were defined as the 2.5th and 97.5th percentiles of the resulting estimates.

One-way sensitivity analyses were conducted to assess the robustness of our projections to key assumptions. First, higher and lower uptake of nirsevimab and RSVPreF was used relative to the main analysis. Second, lower and higher effectiveness estimates were used for both products. Third, the 2019 RSV-attributable mortality estimates were replaced with estimates from the Global Burden of Disease Study 2023 (GBD 2023).^23^ Fourth, the RSV-associated hospitalisation and mortality rates under the no-implementation scenario in 2025 for low-income and lower-middle-income countries were allowed to vary randomly within ±50% of the 2019 estimates, given that evidence of a return towards pre-pandemic RSV activity has primarily come from high-income countries. Further details of the sensitivity analyses are provided in the Appendix (pp 32–34, 55–60).

### Statistical software

All data analyses were done using R software (version 4.3.3).

### Ethics

This modelling study used previously published and publicly available data and did not involve human participants or the collection of identifiable individual-level data. No additional ethics approval was therefore required.

### Patient and public involvement

This study was a modelling analysis of publicly available aggregated data. Patients or members of the public were not involved in the design, conduct, or reporting of this research.

### Role of the funding source

Two co-authors (DRF and ES) from WHO, one of the funders of this study, contributed to the collection of data on RSV prophylactic product use and were involved in study design, data interpretation, and writing of the report. The other funder of the study, National Natural Science Foundation of China, had no role in study design, data collection, data analysis, data interpretation, or writing of the report.

## Results

### The baseline RSV disease burden in 2019

Across the six WHO regions, the African Region had the highest incidence of RSV-associated ALRI in terms of both the incidence rate (117.4 per 1000 person-years, 95% UR: 60.0–229.8) and absolute number (4.3 million episodes, 95% UR: 2.2–8.4, accounting for 37% of the global episodes); the European Region had the lowest incidence rate (53.5 per 1000 person-years, 95% UR: 25.7–111.4). Despite the highest incidence of RSV-associated ALRI, the African Region had the lowest hospital admission rate (12.6 per 1000 person-years; 95% UR: 7.5–21.3), and the Eastern Mediterranean Region exhibited the highest hospital admission rate (29.9 per 1000 person-years, 95% UR: 14.2–63.2) (Table 1 and Appendix p 53).

**Table 1:** Estimated morbidity and mortality burden of RSV in infants in 2019 by WHO regions.

Estimates	AFR	AMR	EMR	EUR	SEAR	WPR
RSV–associated ALRI						
Data points^a^	5 (1)	11 (1)	1 (0)	3 (2)	8 (1)	1 (0)
Rate^b^	117.4 (60.0–229.8)	97.2 (60.9–155.1)	62.5 (41.7–93.7)	53.5 (25.7–111.4)	71.4 (42.5–119.9)	70.9 (53.8–93.4)
N	4,308,000 (2,200,000–8,434,000)	1,386,000 (868,000–2,212,000)	1,138,000 (759,000–1,705,000)	570,000 (274,000–1,187,000)	2,398,000 (1,427,000–4,030,000)	1,563,000 (1,186,000–2,059,000)
RSV–associated ALRI hospital admission						
Data points^a^	20 (1)	30 (3)	2 (0)	27 (7)	12 (3)	16 (4)
Rate^b^	12.6 (7.5–21.3)	21.2 (14.1–31.9)	29.9 (14.2–63.2)	19.2 (15.0–24.6)	16.9 (9.8–29.4)	16.9 (11.3–25.4)
N	463,000 (274,000–782,000)	302,000 (200,000–454,000)	545,000 (258,000–1,151,000)	205,000 (160,000–262,000)	569,000 (328,000–988,000)	373,000 (248,000–560,000)
RSV–associated ALRI in–hospital deaths						
Data points	21	22	10	19	9	15
hCFR (%)	1.54 (0.97–2.31)	0.74 (0.34–1.58)	1.28 (0.61–2.73)	0.16 (0.07–0.38)	1.49 (0.74–3.06)	0.18 (0.04–0.71)
N	7200 (3700–14,000)	2200 (900–5100)	6900 (2600–18,500)	300 (100–800)	8500 (3300–20,300)	700 (100–2600)
RSV–attributable overall deaths						
PAF (%)	2.3 (1.8–3.0)	1.7 (1.3–2.3)	2.1 (1.5–3.2)	1.7 (1.3–2.1)	2.2 (1.4–3.8)	1.8 (1.2–3.0)
N	34,400 (27,000–46,200)	2500 (1900–3200)	9900 (7100–15,000)	1100 (900–1400)	14,200 (9100–25,300)	3200 (2100–5300)
Rate^b^	0.97 (0.76–1.31)	0.17 (0.13–0.22)	0.57 (0.41–0.86)	0.10 (0.08–0.13)	0.41 (0.26–0.74)	0.14 (0.09–0.23)

RSV = respiratory syncytial virus. ALRI = acute lower respiratory infection. AFR = African Region, AMR = Region of the Americas. EMR = Eastern Mediterranean Region. EUR = European Region. SEAR = South-East Asia Region. WPR = Western Pacific Region. N = number of cases. hCFR = in-hospital case fatality ratio. PAF = Population attributable fraction.

a Number in parentheses denotes the number of data points with imputed data.

b Per 1000 person-years.

The RSV hCFR was highest in the African Region (1.54%, 95% UR: 0.97–2.31) and the South-East Asia Region (1.49%, 95% UR: 0.74–3.06). However, in-hospital RSV deaths accounted for only 20.9% of RSV-attributable overall deaths (7200 out of 34,400) in the African Region, equivalent to 79.1% of RSV-attributable deaths occurring out of hospital. The African Region, the South-East Asia Region and the Eastern Mediterranean Region jointly accounted for 88% of the infant RSV-attributable overall deaths. The European Region had the lowest RSV hCFR and RSV-attributable overall mortality rate (Table 1 and Appendix p 53).

The regional variations persisted when further stratifying the estimates above by country income levels. For example, low-income countries in the African Region had much higher RSV-associated ALRI incidence rate and RSV-attributable mortality rate than low-income countries in the South-East Asia Region; upper-middle-income countries in the African Region had higher RSV-associated ALRI incidence rate, hospital admission rate, and mortality rate than upper-middle-income countries in the South-East Asia Region. The only exception was for high-income countries where the incidence rate, hospital admission rate and mortality rate were all comparable between the European Region and Region of the Americas (Fig. 2).

**Fig. 2:**
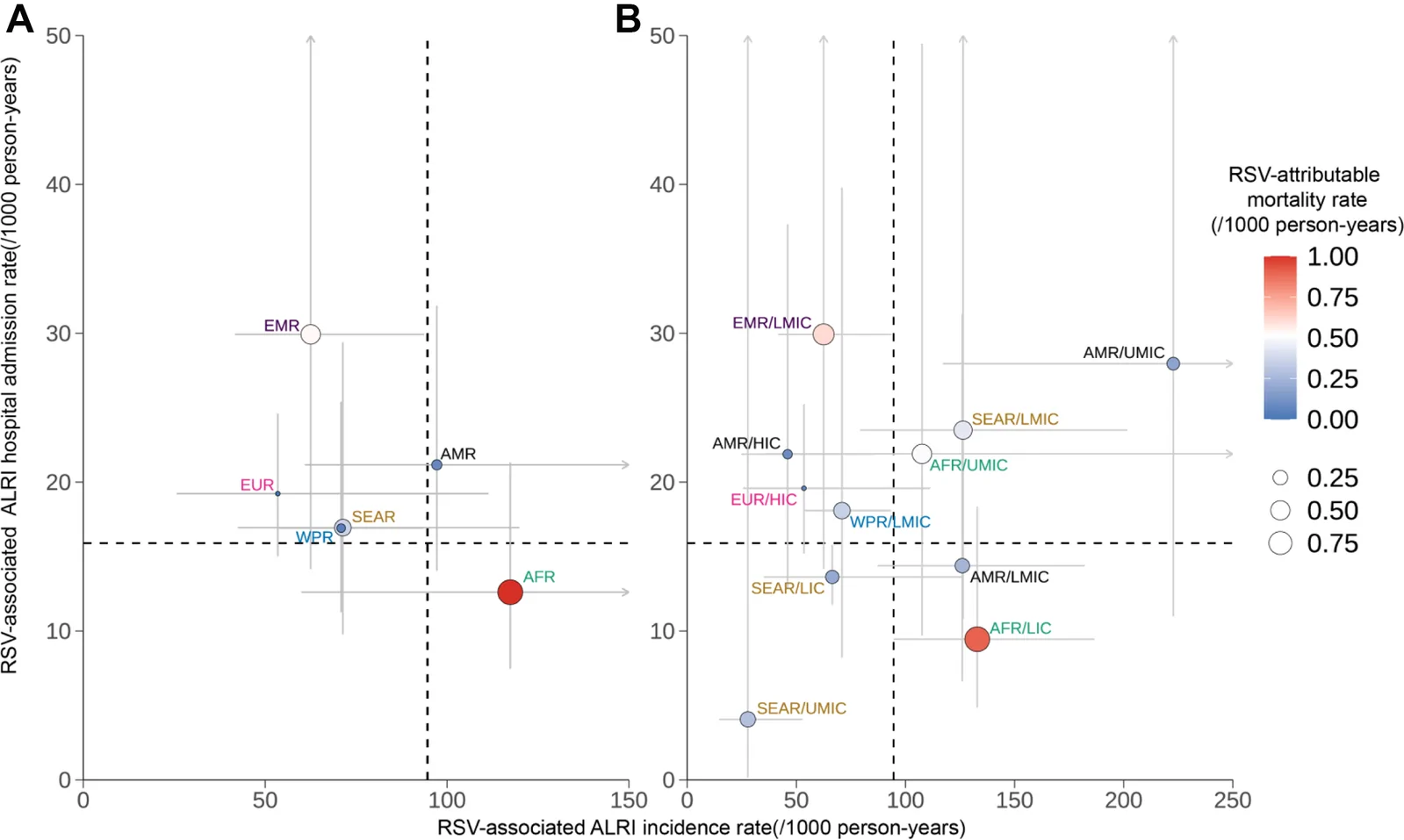
RSV-associated ALRI incidence, hospital admission, and RSV-attributable mortality rates in infants stratified by WHO region (A) and income level within each region (B). Each point represents a regional or region-income group estimate. Circle colour and area represent RSV-attributable mortality rates: white indicates the global average (0.50 per 1000 person-years), red denotes higher rates, and blue denotes lower rates. Horizontal and vertical lines represent the 95% uncertainty range of the RSV-associated incidence and hospital admission rates, respectively. The dashed line represents the global average level based on the previous estimate.^2^ RSV = respiratory syncytial virus. AFR = African Region. AMR = Region of the Americas. EMR = Eastern Mediterranean Region. EUR = European Region. SEAR = South-East Asia Region. WPR = Western Pacific Region. HIC = High-income countries. UMIC = Upper-middle-income countries. LMIC = Lower-middle-income countries. LIC = Low-income countries.

### The projected RSV disease burden

As of June 2025, nirsevimab and RSVpreF had been licensed in 54 and 64 countries, respectively, with 16 and 12 of these countries incorporating them into their NIPs. Most approvals were in the European Region (34 for nirsevimab and 35 for RSVpreF) and the Region of the Americas (8 and 12), followed by the Western Pacific Region (6 and 5) and the Eastern Mediterranean Region (4 and 8). In contrast, approvals were limited in the South-East Asia Region (2 and 2) and the African Region (0 and 2) (Fig. 3). Most countries that licensed nirsevimab (53 of 54) and RSVpreF (59 of 64) were high- or upper-middle-income, and the majority of those incorporating them into NIPs were high-income countries (15 of 16 and 10 of 12, respectively). More detailed information on the licensure status can be found in the Appendix (pp 28–29) and on the developed interactive webpage (https://idem.njmu.edu.cn/shiny/rsvpassiveimmutracker/).

**Fig. 3:**
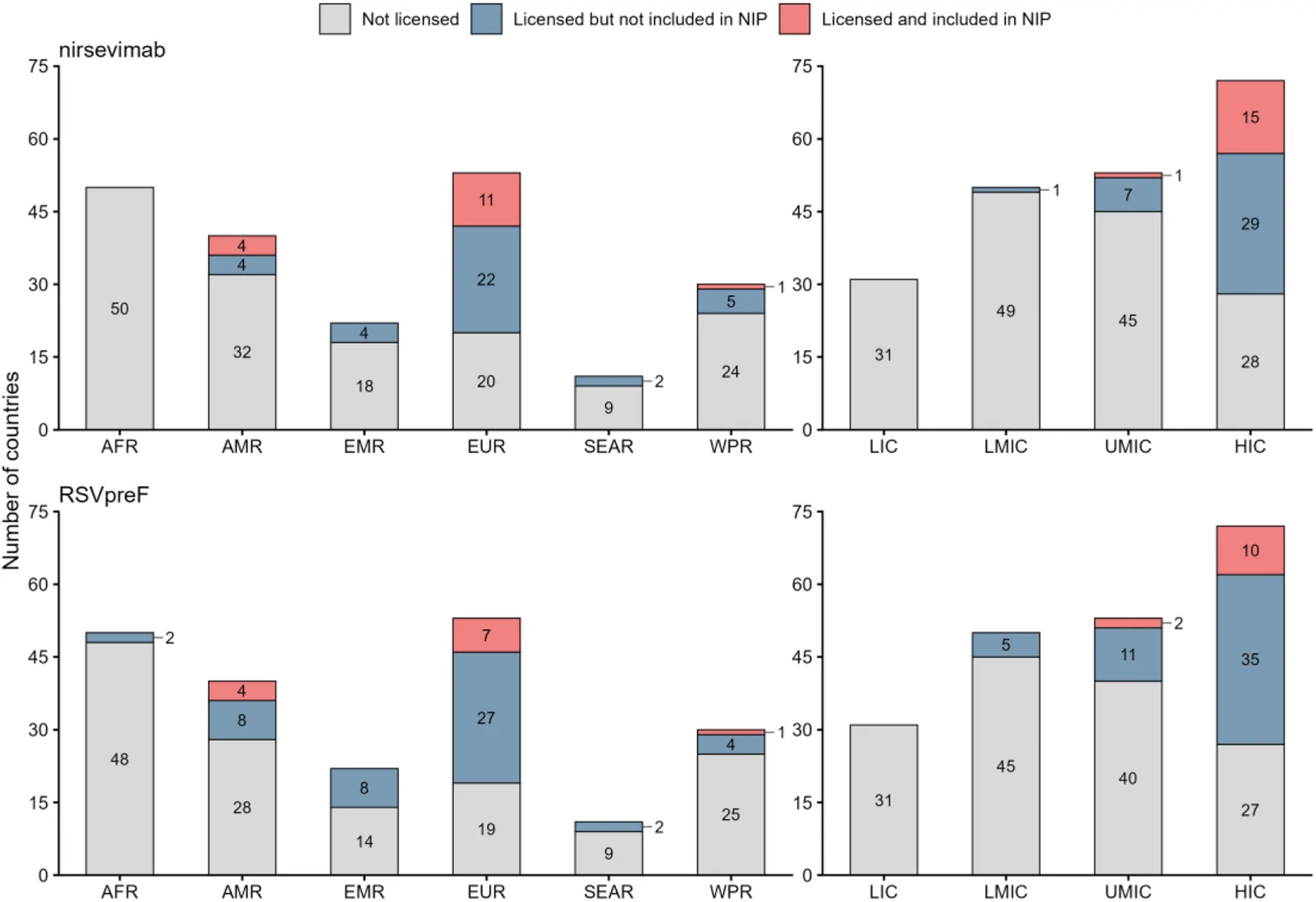
Global implementation landscape of novel RSV prophylactic products for infants, as of June 2025. AFR = African Region. AMR = Region of the Americas. EMR = Eastern Mediterranean Region. EUR = European Region. SEAR = South-East Asia Region. WPR = Western Pacific Region. HIC = High-income countries. UMIC = Upper-middle-income countries. LMIC = Lower-middle-income countries. LIC = Low-income countries. NIP = National Immunisation Program. Detailed information is available at: https://idem.njmu.edu.cn/shiny/rsvpassiveimmutracker.

Based on the licensure status above, we estimated that an annual total of 4,641,000 doses of nirsevimab and 1,955,000 doses of RSVPreF would be administered globally. Compared to the no-implementation scenario, the administration of both products was projected to jointly avert 91,000 (95% UR: 75,000–118,000) RSV-associated ALRI hospital admissions and 300 (95% UR: 210–470) RSV-attributable deaths among infants, corresponding to 3.7% (95% UR: 2.7–4.9) and 0.4% (95% UR: 0.3–0.7) of the total infant RSV admissions and deaths, respectively (Table 2).

**Table 2:** Projected doses of nirsevimab and RSVPreF administered, and RSV-associated ALRI hospital admissions and attributable deaths averted annually in infants under the June-2025 product use scenario, relative to the no-implementation scenario.

	Doses administered		RSV–associated ALRI hospital admissions			RSV–attributable deaths		
	Nirsevimab No. of doses	RSVpreF No. of doses	No implementation^a^ No. of episodes Incidence rate/1000 py	June-2025 product use^a^ No. of episodes Incidence rate/1000 py	Cases averted^a^ No. of episodes Decrease in %	No implementation^a^ No. of deaths Mortality rate/1000 py	June-2025 product use^a^ No. of deaths Morality rate/1000 py	Deaths averted^a^ No. of deaths Decrease in %
By WHO region								
AFR	0	16,000	600,000 (559,000–663,000) 15.2 (14.2–16.8)	600,000 (559,000–663,000) 15.2 (14.2–16.8)	0 (0–0) 0 (0–0.1)	38,600 (30,600–51,400) 1.0 (0.8–1.3)	38,600 (30,600–51,400) 1.0 (0.8–1.3)	0 (0–10) 0 (0–0)
AMR	2,454,000	823,000	351,000 (264,000–503,000) 26.6 (20.0–38.1)	297,000 (221,000–445,000) 22.5 (16.8–33.7)	52,000 (36,000–79,000) 14.7 (9.8–21.2)	2200 (1700–2900) 0.2 (0.1–0.2)	2100 (1600–2700) 0.2 (0.1–0.2)	180 (100–350) 8.0 (4.8–13.7)
EMR	7000	88,000	438,000 (318,000–688,000) 22.7 (16.5–35.6)	437,000 (317,000–687,000) 22.6 (16.4–35.5)	1000 (1000–2000) 0.3 (0.3–0.3)	11,700 (8400–17,700) 0.6 (0.4–0.9)	11,600 (8400–17,600) 0.6 (0.4–0.9)	20 (10–30) 0.2 (0.1–0.2)
EUR	2,011,000	740,000	157,000 (147,000–169,000) 16.5 (15.5–17.7)	124,000 (115,000–133,000) 13.0 (12.1–14.0)	34,000 (31,000–37,000) 21.4 (19.8–22.9)	1000 (800–1300) 0.1 (0.1–0.1)	1000 (800–1200) 0.1 (0.1–0.1)	70 (50–110) 7.2 (4.8–10.7)
SEAR	14,000	50,000	708,000 (415,000–1,451,000) 21.4 (12.5–43.8)	707,000 (415,000–1,450,000) 21.4 (12.5–43.8)	0 (0–2000) 0 (0–0.2)	13,700 (8700–24,400) 0.4 (0.3–0.7)	13,700 (8700–24,400) 0.4 (0.3–0.7)	10 (10–20) 0.1 (0–0.1)
WPR	154,000	238,000	150,000 (123,000–199,000) 10.3 (8.4–13.6)	147,000 (119,000–196,000) 10.1 (8.2–13.4)	3000 (3000–4000) 2.2 (1.6–3.0)	2300 (1700–3500) 0.2 (0.1–0.2)	2300 (1600–3400) 0.2 (0.1–0.2)	10 (10–20) 0.4 (0.3–0.8)
By income level								
HIC	4,453,000	1,580,000	244,000 (209,000–304,000) 20.8 (17.9–26.0)	147,000 (119,000–196,000) 10.1 (8.2–13.4)	81,000 (66,000–108,000) 33.2 (31.0–35.9)	800 (600–1100) 0.1 (0.0–0.1)	600 (400–800) 0.0 (0.0–0.1)	220 (140–390) 29.1 (22.6–35.2)
UMIC	176,000	317,000	446,000 (342,000–858,000) 15.4 (11.8–29.5)	439,000 (336,000–849,000) 15.1 (11.6–29.2)	8000 (5000–15,000) 1.7 (1.0–3.2)	5700 (4500–7400) 0.2 (0.1–0.2)	5600 (4400–7300) 0.2 (0.1–0.2)	50 (40–80) 0.9 (0.7–1.4)
LMIC	11,000	59,000	1,463,000 (1,133,000–2,098,000) 23.4 (18.1–33.6)	1,462,000 (1,133,000–2,097,000) 23.4 (18.1–33.5)	1000 (1000–2000) 0.1 (0–0.1)	41,100 (30,700–56,000) 0.7 (0.5–0.9)	41,100 (30,700–56,000) 0.7 (0.5–0.9)	20 (10–30) 0.1 (0–0.1)
LIC	0	0	260,000 (219,000–322,000) 10.0 (8.4–12.4)	260,000 (219,000–322,000) 10.0 (8.4–12.4)	0 (0–0) 0 (0–0)	22,900 (18,900–28,600) 0.9 (0.7–1.1)	22,900 (18,900–28,600) 0.9 (0.7–1.1)	0 (0–0) 0 (0–0)
Global	4,641,000	1,955,000	2,446,000 (2,070,000–3,200,000) 18.9 (16.0–24.8)	2,352,000 (1,984,000–3,121,000) 18.2 (15.3–24.1)	91,000 (75,000–118,000) 3.7 (2.7–4.9)	70,700 (59,000–86,400) 0.6 (0.5–0.7)	70,300 (58,700–86,100) 0.5 (0.4–0.7)	300 (210–470) 0.4 (0.3–0.7)

No. = number; py = person-years. AFR = African Region. AMR = Region of the Americas. EMR = Eastern Mediterranean Region. EUR = European Region. SEAR = South-East Asia Region. WPR = Western Pacific Region. HIC = High-income countries. UMIC = Upper-middle-income countries. LMIC = Lower-middle-income countries. LIC = Low-income countries.

a Values in parentheses indicate the 95% uncertainty range.

Across the six WHO regions, the largest absolute number of averted hospital admissions was in the Region of the Americas (52,000, 95% UR: 36,000–79,000) while the largest relative proportion of averted hospital admissions was in the European Region (21.4%, 95% UR: 19.8–22.9). For mortality, the largest absolute number of averted deaths was observed in the Region of the Americas (180, 95% UR: 100–350), which also accounted for the largest relative proportion (8.0%, 95% UR: 4.8–13.7) (Table 2).

Globally, approximately 90% of averted hospital admissions and over 70% of averted deaths occurred in infants in high-income countries (81,000 [95% UR: 66,000–108,000] and 220 [95% UR: 140–390], respectively), corresponding to reductions of 33.2% (95% UR 31.0–35.9) and 29.1% (95% UR 22.6–35.2); by contrast, the relative reduction of admissions and deaths in middle-income countries were less than 5%, and no reduction was observed in low-income countries (Table 2).

Sensitivity analyses under more conservative or optimistic assumptions regarding overall uptake and prophylactic product effectiveness showed trends consistent with the main analysis. Allowing variation in 2025 RSV-associated hospitalisation and mortality rates resulted in wider uncertainty ranges but did not materially alter the projected impacts. Using GBD 2023 mortality estimates as an alternative data source similarly yielded results consistent with the main analysis (Appendix pp 55–60).

### Regional inequality

Globally, marked regional disparities were observed in the distribution of RSV-attributable deaths under the no-implementation scenario, with the Q5-Q1 ratio of 18.3 (95% UR: 11.7–27.5), indicating that the risk of RSV mortality was over 18 times greater in the highest-risk quintile of the population compared to the lowest-risk quintile. Under the June-2025 product use scenario, the Q5-Q1 ratio showed a possible small increase to 20.8 (95% UR: 13.0–31.7). Within WHO regions, the most pronounced increase was found in the Region of the Americas (from 5.8 [95% UR 3.4–13.6] to 10.4 [95% UR 5.0–25.2]) and the European Region (from 12.7 [95% UR 8.0–20.0] to 22.1 [95% UR 13.1–35.7]) (Fig. 4). Sensitivity analyses varying prophylactic product uptake and effectiveness, as well as allowing RSV-associated hospitalisation and mortality rates under the 2025 no-implementation scenario to vary, showed a consistent increase in the median Q5-Q1 ratio compared with the main analysis (Figure S19). Sensitive analyses using the GBD 2023 mortality data showed a similar increasing trend of the Q5-Q1 ratio as observed in the main analysis, although both the absolute values and the magnitude of increases were larger than the main analysis (Figures S16 and S17). Furthermore, compared with the main analysis, the percentage reduction in RSV-attributable mortality under the June-2025 product use scenario when using the GBD 2023 mortality data was even lower (Figure S18).

**Fig. 4:**
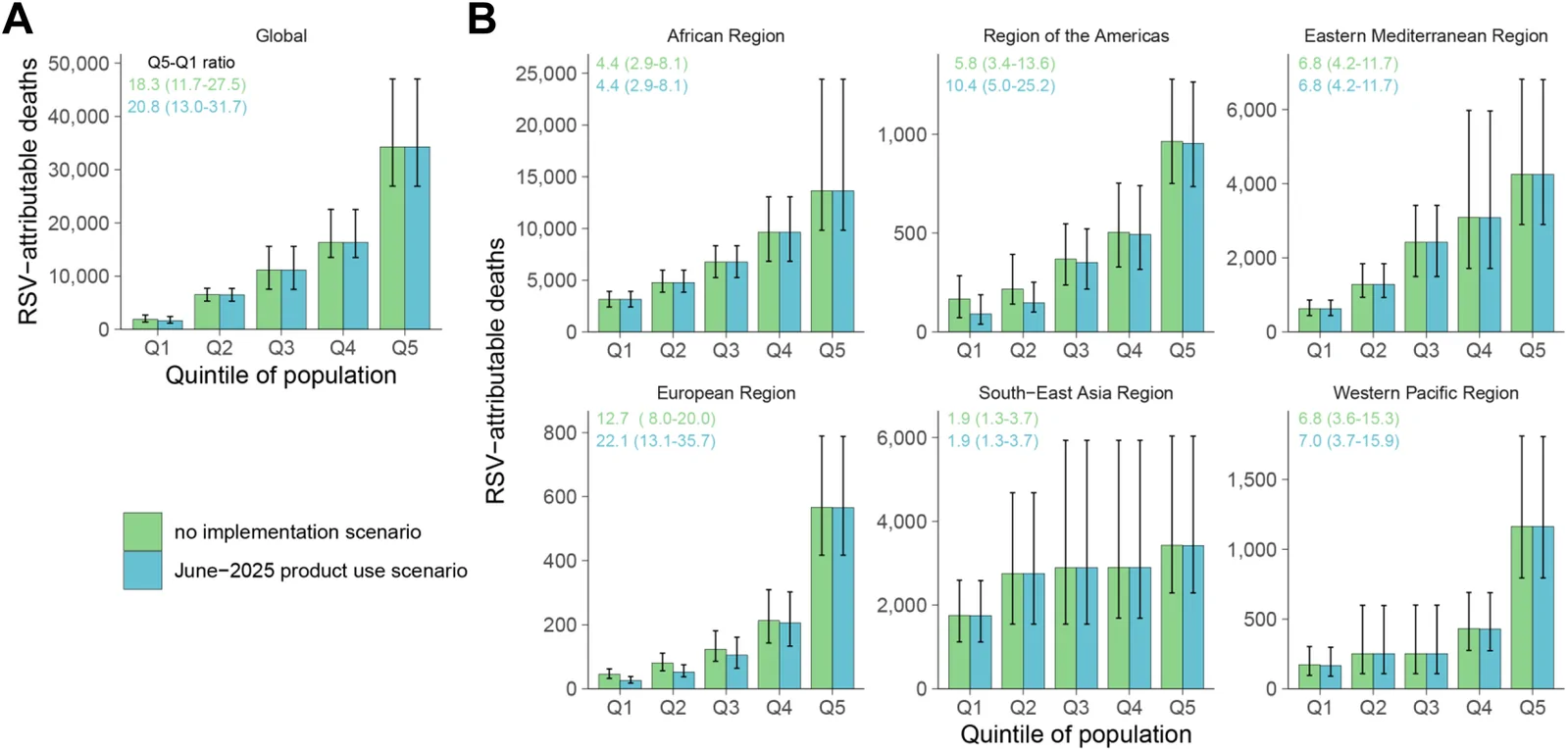
Projected annual RSV-attributable deaths in infants by population quintile under no-implementation scenario and June-2025 product use scenario globally (panel A) and by WHO region (panel B). Error bars represent the 95% uncertainty range. Numbers in the top-left corner of each panel indicate the ratio of RSV-attributable deaths in the fifth quintile to those in the first quintile (Q5-Q1 ratio), with higher values reflecting greater disparities in RSV mortality.

## Discussion

In this study, we estimated the baseline burden of RSV disease among infants in 2019 and showed substantial disparities in RSV morbidity and mortality burden across WHO regions and country income levels. Specifically, the African region had the highest RSV-associated ALRI incidence rate and RSV-attributable mortality rate, but the lowest RSV-associated ALRI hospital admission rate, resulting a high out-of-hospital RSV mortality burden, which accounted for 79% (95% UR: 59–90) of overall RSV-attributable deaths; 90% (95% UR: 86–92) of RSV-attributable deaths were concentrated in the African, South-East Asia, and Eastern Mediterranean regions. However, as of June 2025, countries that introduced or licensed novel RSV prophylactic products were primarily from the European and Americas Region (high-income countries with least RSV morbidity and mortality burden). As a result, our projection showed only a modest reduction globally in the infant RSV hospitalisation and mortality burden with the current implementation status of these prophylactic products, while the existing inequalities in RSV mortality burden were projected to increase, with the mortality risk ratio between the highest-risk quintile and the lowest-risk quintile increasing from 18.3 to 20.8.

Despite the fact that RSV infects almost all infants before their second birthday in all countries, the excess burden of severe ALRI morbidity and death is highest in the poorest regions and countries. Compared to the previously published global RSV disease burden estimates,^2^ we showed that further stratification by WHO region revealed two distinct patterns of the baseline RSV-associated ALRI morbidity and mortality burden in 2019. The European region and high-income countries in the Region of the Americas showed lower RSV-associated ALRI incidence and RSV-attributable overall mortality in the community, but comparatively higher hospital admission rates. Conversely, low-income countries in the African and South-East Asian regions demonstrated higher RSV-associated ALRI incidence and RSV-attributable mortality but disproportionately lower RSV-associated hospital admission rates. The observed disparity in hospitalisation rates is unlikely to reflect lower disease severity, as the incidence and overall mortality in these regions were relatively higher. Instead, it is consistent with limited healthcare access due to affordability barriers, as well as the distance and time to reach medical care.^24^^,^^25^ The most severe outcomes of RSV-associated ALRI, such as death, are treatable by supportive care, including supplemental oxygen and intravenous hydration. Children died of RSV-associated ALRI in the community because they are unable to access this life-saving supportive care in time. Moreover, in these resource-limited settings, even those children who do make it to hospital might be turned away due to hospital bed shortages or receive inadequate care once admitted, as a result of overcrowding and constrained resources. A recent study in Bangladesh reported that 18.4% of children requiring hospitalisation were denied admission due to insufficient bed capacity, with a corresponding hazard ratio for death of 1.56 (95% CI 1.34–1.81) compared with admitted children.^26^ This highlights the shortage of healthcare resources in resource-limited countries but also implies that implementing RSV preventive interventions in these settings could achieve greater impact than in well-resourced settings as these interventions might decrease excess out-of-hospital mortality caused by the inability to access necessary medical care.

Only modest reductions in the infant RSV hospitalisation and mortality burden were anticipated globally with the current implementation of novel RSV prophylactic products, accounting for to 3.7% (95% UR: 2.7–4.9) and 0.4% (0.3–0.7) of the total RSV admissions and deaths, respectively. The largest decreases were observed in high-income countries, which were projected to see a reduction of 33.2% (31.0–35.9) and 29.1% (22.6–35.2) of the RSV admissions and deaths in these countries. However, these large reductions in proportion could not be translated into large reduction in absolute number of episodes globally as uptake remained low in countries bearing high disease burden; middle-income countries, with limited uptake of RSV prophylactic products, were projected to see a much smaller reduction (<5% in both RSV admissions or deaths) and low-income countries, with no uptake of RSV preventive products, would see no reductions.

Our estimated impact of RSV prophylactic products was broadly comparable to that reported in recent modelling and real-world studies. Menegale et al. estimated in a modelling study that routine seasonal monoclonal antibody administration to 80% of newborns could avert 50.2% (95% CI 43.5–55.8) of RSV hospitalisations in the total population during the 2024–25 RSV season in Lombardy, Italy.^27^ Similarly, we estimated that implementation of RSV prophylactic products with 69% coverage could avert 44.5% (95% UR 43.7–45.3) of RSV-associated hospital admissions among infants in Italy. In a modelling study in the US, Yarnoff et al. estimated that 44.7% nirsevimab coverage during the 2024–25 RSV season could avert 18,765 hospitalisations. With a higher coverage of 51%, our model estimated that 24,729 (95% UR 14,777–42,542) hospitalisations could be averted in the US.^28^ Using real-world data, Perramon-Malavez et al. reported that approximately 90% coverage among infants aged 0–<6 months in Catalonia, Spain, was associated with a 60% (95% CI 57–63) reduction in bronchiolitis hospital admissions during the 2023–24 RSV season^29^; by comparison, our estimated reduction in RSV-associated ALRI hospital admissions in Spain in 2025 was somewhat higher (74.3% [95% UR 73.8–74.5]); this apparently higher estimate was as expected given that we used RSV-specific ALRI rather than all-cause bronchiolitis.

Due to the wider implementation of RSV prophylactic products in regions with relatively low baseline RSV-associated ALRI incidence and RSV-attributable mortality compared to those bearing higher disease burden, global inequalities in RSV disease burden would widen, as evidenced by the increasing Q5-Q1 ratio for RSV-attributable mortality. Globally, compared to the no-implementation scenario, the Q5-Q1 ratio for RSV-attributable mortality was projected to increase from 18.3 to 20.8 under the June-2025 product use scenario; the increase was particularly substantial in regions with several countries introducing RSV prophylactic products, such as the Regions of the Americas (from 5.8 to 10.4), and the European regions (from 12.7 to 22.1). Lessons learnt from the introduction and implementation of pneumococcal and rotavirus vaccines suggest that substantial disparities in coverage between LMICs and high-income countries could last for several years after initial vaccine introductions^30^; as the implementation was expected to constantly scale up in high-income countries, the global equality in the infant RSV disease burden could worsen even further than our projection in the next 5 years.

The findings of this study underscore the urgent need for a coordinated multi-stakeholder effort to accelerate the approval, introduction, and scale-up of novel RSV prophylactic products in high RSV disease burden settings. Recent global advancements, such as WHO SAGE's recommendation on infant RSV passive immunisation in the end of 2024,^31^ WHO prequalification of the maternal vaccine in March 2025,^32^ and Gavi's approval of a funding window for RSV maternal vaccination programmes in July 2025,^33^ will likely accelerate access in LMICs, although still lagging several years behind introduction in HICs. To address affordability challenges, strategies such as pooled procurement through UNICEF- and PAHO-led initiatives,^34^ and the development of more affordable formulations, such as the Gates Foundation-supported multi-dose vial (MDV) RSV maternal vaccine offer viable approaches.^35^ Additional strategies include tiered pricing, technology transfer, and fostering market competition.^36^, ^37^, ^38^ For non-Gavi-eligible LMICs, strengthening National Immunization Technical Advisory Groups (NITAGs), increasing immunisation budgets, and conducting cost-effectiveness studies are essential to inform policy and facilitate NIP inclusion.^39^, ^40^, ^41^ Beyond financing and policy, addressing evidence and awareness gaps on RSV remains crucial. Strengthening RSV surveillance and real-world effectiveness studies in LMICs will build locally relevant evidence for informed decision-making.

Our study had several limitations. First, we assumed that in the absence of RSV prophylactic products, RSV disease burden and seasonality would have broadly returned to pre-pandemic patterns in 2025; therefore, we based our projections on 2019 RSV burden estimates rather than data from the pandemic and post-pandemic period, as RSV epidemiology was temporarily disrupted by the COVID-19 pandemic and associated public health measures.^42^^,^^43^ Although this assumption is supported by recent studies and surveillance data from multiple countries showing a broad return of RSV activity towards pre-pandemic patterns,^16^, ^17^, ^18^, ^19^, ^20^, ^21^ the available evidence might not fully represent all countries, as much of it came from high-income settings. Sensitivity analyses allowing 2025 morbidity and mortality rates to vary around pre-pandemic levels, as well as analyses based on GBD 2023 mortality data, yielded results consistent with the main analysis, suggesting that our findings were robust to variations in the assumed 2025 morbidity and mortality rates. Second, we used a static model, which did not account for indirect protection, rather than a dynamic transmission model for projecting the impact of the implementation of the novel prophylactic products. Nonetheless, existing evidence suggests that static and dynamic models generally produced similar estimates of RSV-related hospitalisations and deaths averted since herd immunity was limited due to a small proportion of the general population being immunised (i.e., the infant population)^44^; moreover, existing evidence did not suggest the effect of preventing transmission by nirsevimab.^45^ Third, because data on nirsevimab and maternal RSVpreF vaccine uptake were unavailable for most countries, we assumed uptake based on the implementation status of these prophylactic products or, for maternal vaccination, using coverage of a similar vaccine (Tdap) as a reference. Although these assumptions may differ from real-world uptake, sensitivity analysis results with varied uptake scenarios generally yielded similar trends, despite variations in absolute impact, as expected. Fourth, our estimates did not capture the burden of RSV-associated upper respiratory infections (URIs) occurring without progression to more severe disease, which constitute part of the broader spectrum of RSV morbidity. We focused on more severe RSV outcomes, including RSV-associated ALRI hospital admissions and deaths, which are the primary outcomes targeted by the current RSV immunoprophylactic products.^4^^,^^5^ Finally, the licensing and implementation of RSV prophylactic products is a dynamic process, and the implementation status used in our analysis, based on data up to June 2025, may not fully reflect the situation in the upcoming years. In addition, although clesrovimab received the US Advisory Committee on Immunization Practices (ACIP) recommendation as an alternative to nirsevimab in June 2025,^46^ no real-world effectiveness data are currently available, and we therefore did not consider its use in our projections. Nonetheless, as both long-acting monoclonal antibody products have demonstrated comparable efficacy against RSV-associated hospitalisation,^47^^,^^48^ we expect that the substitution of clesrovimab for nirsevimab in some settings would shift product-specific uptake without substantially altering the projected population-level protection.

Global inequality in the infant RSV disease burden has long existed, and the current inequitable rollout of RSV prophylactic products may exacerbate this disparity. Although meaningful progress is being made towards the introduction of these products to high-disease-burden settings, substantial challenges persist. At this critical juncture for RSV prevention, coordinated efforts by international agencies, governments, manufacturers, researchers, and the public are essential to overcome existing barriers and jointly accelerate the introduction and scale-up of infant RSV immunisation programmes in low- and middle-income countries, thereby promoting global health equity.

## Contributors

YL conceptualised the study with inputs from DRF and ES. SR led data collection with inputs from BC and JZ. BC and SR co-led data analysis with inputs from LG. YL led data interpretation with inputs from HN, DRF, ES, SR and BC. SR wrote the first draft with inputs from YL, BC, HN, ES, and DRF. All authors reviewed the manuscript for intellectual content, and approved the final draft for submission. SR, BC, and YL had full access to and verified the aggregated study data for analysis in this study, and had final responsibility for the decision to submit for publication.

## Data sharing statement

All study data included in the analysis are in the public domain and have been properly cited.

## Editor note

The Lancet Group takes a neutral position with respect to territorial claims in published maps and institutional affiliations.

## Declaration of interests

HN reports grants from WHO, Innovative Medicines Initiative, the National Institute for Health Research, Pfizer, and Icosavax; and personal fees from the Gates Foundation, Pfizer, ReViral, GSK, Merck, Icosavax, Sanofi, Novavax and AbbVie, outside the submitted work. YL reports consultancy fees from WHO, related to the submitted work; grants from GSK, Pfizer, MSD and WHO to institution, consultancy fees from Pfizer, GSK, MSD and WHO, outside the submitted work. All other authors declare no competing interests.
