# Supplementary material for: Regional inequality in infant RSV morbidity and mortality burden in the era of expanded RSV passive immunisation: a global projection modelling study
Source: eClinicalMedicine. 2026 Sep 23;100:104219. doi: 10.1016/j.eclinm.2026.104219 (PMC13634261; doi:10.1016/j.eclinm.2026.104219)
Supplement: Supplementary file 1 [file mmc1.docx]

**Supplemental Appendix**

Regional inequality in infant RSV morbidity and mortality burden in the era of expanded RSV passive immunisation: a global projection modelling study

**Contents**

[Supplementary methods	3](#_Toc213406185)

[List of countries by WHO region and World Bank income level (2019) in the analysis	3](#_Toc213406186)

[Imputation method for incidence rate estimation in 0–<12 months	9](#_Toc213406187)

[Method of estimating RSV disease burden in 2019	10](#_Toc213406188)

[Geographical distribution of data included for disease burden estimation	12](#_Toc213406189)

[Description of individual data points included for disease burden estimation	15](#_Toc213406190)

[List of countries with nirsevimab or RSVpreF licensure or NIP inclusion as of June 2025	28](#_Toc213406191)

[Country-level uptake of nirsevimab and RSVPreF	30](#_Toc213406192)

[Method of estimating the proportions of RSV-ALRI hospital admissions by age in months and by birth month	35](#_Toc213406193)

[Method of determining the immunisation timing of individual countries	42](#_Toc213406194)

[Method of projecting RSV-associated hospital admissions and mortality burden	47](#_Toc213406195)

[Supplementary results	53](#_Toc213406196)

[Comparison of baseline RSV morbidity and mortality burden in infants in 2019 across WHO regions.	53](#_Toc213406197)

[A ShinyApp tracking the implementation status of global RSV prevention products for infants	54](#_Toc213406198)

[Results of sensitivity analyses	55](#_Toc213406199)

[References	60](#_Toc213406200)

Supplementary methods

List of countries by WHO region and World Bank income level (2019) in the analysis

**Table S1.** List of countries by WHO region and World Bank income level (2019) in the analysis.

| Country | WHO Region | Income level |
| --- | --- | --- |
| Afghanistan | Eastern Mediterranean Region | Low |
| Albania | European Region | Upper-middle |
| Algeria | African Region | Lower-middle |
| Andorra | European Region | High |
| Angola | African Region | Lower-middle |
| Antigua and Barbuda | Region of the Americas | High |
| Argentina | Region of the Americas | Upper-middle |
| Armenia | European Region | Upper-middle |
| Aruba | Region of the Americas | High |
| Australia | Western Pacific Region | High |
| Austria | European Region | High |
| Azerbaijan | European Region | Upper-middle |
| Bahamas | Region of the Americas | High |
| Bahrain | Eastern Mediterranean Region | High |
| Bangladesh | South-East Asian Region | Lower-middle |
| Barbados | Region of the Americas | High |
| Belarus | European Region | Upper-middle |
| Belgium | European Region | High |
| Belize | Region of the Americas | Upper-middle |
| Benin | African Region | Lower-middle |
| Bhutan | South-East Asian Region | Lower-middle |
| Bolivia (Plurinational State of) | Region of the Americas | Lower-middle |
| Bosnia and Herzegovina | European Region | Upper-middle |
| Botswana | African Region | Upper-middle |
| Brazil | Region of the Americas | Upper-middle |
| Brunei Darussalam | Western Pacific Region | High |
| Bulgaria | European Region | Upper-middle |
| Burkina Faso | African Region | Low |
| Burundi | African Region | Low |
| Cambodia | Western Pacific Region | Lower-middle |
| Cameroon | African Region | Lower-middle |
| Canada | Region of the Americas | High |
| Cape Verde | African Region | Lower-middle |
| Central African Republic | African Region | Low |
| Chad | African Region | Low |
| Chile | Region of the Americas | High |
| China | Western Pacific Region | Upper-middle |
| Colombia | Region of the Americas | Upper-middle |
| Comoros | African Region | Lower-middle |
| Congo | African Region | Lower-middle |
| Cook Islands | Western Pacific Region | High |
| Costa Rica | Region of the Americas | Upper-middle |
| Croatia | European Region | High |
| Cuba | Region of the Americas | Upper-middle |
| Cyprus | European Region | High |
| Czech Republic | European Region | High |
| Côte d'Ivoire | African Region | Lower-middle |
| Democratic People's Republic of Korea | South-East Asian Region | Low |
| Democratic Republic of the Congo | African Region | Low |
| Denmark | European Region | High |
| Djibouti | Eastern Mediterranean Region | Lower-middle |
| Dominica | Region of the Americas | Upper-middle |
| Dominican Republic | Region of the Americas | Upper-middle |
| Ecuador | Region of the Americas | Upper-middle |
| Egypt | Eastern Mediterranean Region | Lower-middle |
| El Salvador | Region of the Americas | Lower-middle |
| Equatorial Guinea | African Region | Upper-middle |
| Eritrea | African Region | Low |
| Estonia | European Region | High |
| Ethiopia | African Region | Low |
| Fiji | Western Pacific Region | Upper-middle |
| Finland | European Region | High |
| France | European Region | High |
| French Guiana | Region of the Americas | High |
| French Polynesia | Western Pacific Region | High |
| Gabon | African Region | Upper-middle |
| Gambia | African Region | Low |
| Georgia | European Region | Upper-middle |
| Germany | European Region | High |
| Ghana | African Region | Lower-middle |
| Greece | European Region | High |
| Grenada | Region of the Americas | Upper-middle |
| Guadeloupe | Region of the Americas | High |
| Guam | Western Pacific Region | High |
| Guatemala | Region of the Americas | High |
| Guinea | African Region | Low |
| Guinea-Bissau | African Region | Low |
| Guyana | Region of the Americas | Upper-middle |
| Haiti | Region of the Americas | Low |
| Honduras | Region of the Americas | Lower-middle |
| Hungary | European Region | High |
| Iceland | European Region | High |
| India | South-East Asian Region | Lower-middle |
| Indonesia | South-East Asian Region | Upper-middle |
| Iran | Eastern Mediterranean Region | Upper-middle |
| Iraq | Eastern Mediterranean Region | Upper-middle |
| Ireland | European Region | High |
| Israel | European Region | High |
| Italy | European Region | High |
| Jamaica | Region of the Americas | Upper-middle |
| Japan | Western Pacific Region | High |
| Jordan | Eastern Mediterranean Region | Upper-middle |
| Kazakhstan | European Region | Upper-middle |
| Kenya | African Region | Lower-middle |
| Kiribati | Western Pacific Region | Lower-middle |
| Kuwait | Eastern Mediterranean Region | High |
| Kyrgyzstan | European Region | Lower-middle |
| Lao People's Democratic Republic | Western Pacific Region | Lower-middle |
| Latvia | European Region | High |
| Lebanon | Eastern Mediterranean Region | Upper-middle |
| Lesotho | African Region | Lower-middle |
| Liberia | African Region | Low |
| Libya | Eastern Mediterranean Region | Upper-middle |
| Lithuania | European Region | High |
| Luxembourg | European Region | High |
| Madagascar | African Region | Low |
| Malawi | African Region | Low |
| Malaysia | Western Pacific Region | Upper-middle |
| Maldives | South-East Asian Region | Upper-middle |
| Mali | African Region | Low |
| Malta | European Region | High |
| Marshall Islands | Western Pacific Region | Upper-middle |
| Martinique | Region of the Americas | High |
| Mauritania | African Region | Lower-middle |
| Mauritius | African Region | High |
| Mayotte | African Region | High |
| Mexico | Region of the Americas | Upper-middle |
| Micronesia (Federated States of) | Western Pacific Region | Lower-middle |
| Monaco | European Region | High |
| Mongolia | Western Pacific Region | Lower-middle |
| Montenegro | European Region | Upper-middle |
| Morocco | Eastern Mediterranean Region | Lower-middle |
| Mozambique | African Region | Low |
| Myanmar | South-East Asian Region | Lower-middle |
| Namibia | African Region | Upper-middle |
| Nauru | Western Pacific Region | High |
| Nepal | South-East Asian Region | Lower-middle |
| Netherlands | European Region | High |
| New Caledonia | Western Pacific Region | High |
| New Zealand | Western Pacific Region | High |
| Nicaragua | Region of the Americas | Lower-middle |
| Niger | African Region | Low |
| Nigeria | African Region | Lower-middle |
| Niue | Western Pacific Region | Low |
| Norway | European Region | High |
| Occupied Palestinian Territory | Eastern Mediterranean Region | Lower-middle |
| Oman | Eastern Mediterranean Region | High |
| Pakistan | Eastern Mediterranean Region | Lower-middle |
| Palau | Western Pacific Region | High |
| Panama | Region of the Americas | High |
| Papua New Guinea | Western Pacific Region | Lower-middle |
| Paraguay | Region of the Americas | Upper-middle |
| Peru | Region of the Americas | Upper-middle |
| Philippines | Western Pacific Region | Lower-middle |
| Poland | European Region | High |
| Portugal | European Region | High |
| Puerto Rico | Region of the Americas | High |
| Qatar | Eastern Mediterranean Region | High |
| Republic of Korea | Western Pacific Region | High |
| Republic of Moldova | European Region | Lower-middle |
| Reunion | African Region | High |
| Romania | European Region | High |
| Russian Federation | European Region | Upper-middle |
| Rwanda | African Region | Low |
| Saint Kitts and Nevis | Region of the Americas | High |
| Saint Lucia | Region of the Americas | Upper-middle |
| Saint Vincent and the Grenadines | Region of the Americas | Upper-middle |
| Samoa | Western Pacific Region | Upper-middle |
| San Marino | European Region | High |
| Sao Tome and Principe | African Region | Lower-middle |
| Saudi Arabia | Eastern Mediterranean Region | High |
| Senegal | African Region | Lower-middle |
| Serbia | European Region | Upper-middle |
| Seychelles | African Region | High |
| Sierra Leone | African Region | Low |
| Singapore | Western Pacific Region | High |
| Slovakia | European Region | High |
| Slovenia | European Region | High |
| Solomon Islands | Western Pacific Region | Lower-middle |
| Somalia | Eastern Mediterranean Region | Low |
| South Africa | African Region | Upper-middle |
| South Sudan | African Region | Low |
| Spain | European Region | High |
| Sri Lanka | South-East Asian Region | Lower-middle |
| Sudan | Eastern Mediterranean Region | Low |
| Suriname | Region of the Americas | Upper-middle |
| Swaziland | African Region | Lower-middle |
| Sweden | European Region | High |
| Switzerland | European Region | High |
| Syrian Arab Republic | Eastern Mediterranean Region | Low |
| Tajikistan | European Region | Low |
| Thailand | South-East Asian Region | Upper-middle |
| The former Yugoslav Republic of Macedonia | European Region | Upper-middle |
| Timor-Leste | South-East Asian Region | Lower-middle |
| Togo | African Region | Low |
| Tonga | Western Pacific Region | Upper-middle |
| Trinidad and Tobago | Region of the Americas | High |
| Tunisia | Eastern Mediterranean Region | Lower-middle |
| Turkey | European Region | Upper-middle |
| Turkmenistan | European Region | Upper-middle |
| Tuvalu | Western Pacific Region | Upper-middle |
| USA | Region of the Americas | High |
| Uganda | African Region | Low |
| Ukraine | European Region | Lower-middle |
| United Arab Emirates | Eastern Mediterranean Region | High |
| United Kingdom | European Region | High |
| United Republic of Tanzania | African Region | Lower-middle |
| Uruguay | Region of the Americas | High |
| Uzbekistan | European Region | Lower-middle |
| Vanuatu | Western Pacific Region | Lower-middle |
| Venezuela (Bolivarian Republic of) | Region of the Americas | Upper-middle |
| Viet Nam | Western Pacific Region | Lower-middle |
| Western Sahara | African Region | Low |
| Yemen | Eastern Mediterranean Region | Low |
| Zambia | African Region | Lower-middle |
| Zimbabwe | African Region | Lower-middle |

Imputation method for incidence rate estimation in 0–<12 months

**Overview**

Several data points in the global RSV disease burden dadaset^1^ did not report specific incidence rate data for infants but instead provided burden estimates for broader age groups (e.g., 0–<24 months, 0–<36 months, and 0–<60 months). To maximise data utilisation and improve geographical representation, we imputed the missing incidence rate data for the 0–<12 months group using data from the 0–<24 months, 0–<36 months, and 0–<60 months groups as reported in these studies.

**Method**

We considered a multiple imputation approach as detailed previously.^1,2^ We first conducted a meta-analysis (through generalised linear mixed-effect model, GLMM) of the incidence (or hospitalisation) rate ratio (IRR) between 0–<12 months and each of the three age groups, 0–<24 months, 0–<36 months, and 0–<60 months, using all data sources that had available data for both age groups. This resulted in both the point estimate and the standard error of the meta-estimated IRR (**Table S2**). Subsequently, we generated 1 000 samples of IRR and estimated 1 000 samples of the incidence or hospitalisation rate for 0–<12 months.

**Table S2.** Incidence rate ratio (95% confidence interval) between 0–<12 months and each of the three age groups (0–<24, 0–<36, and 0–<60 months).

| Age group pair | IRR for  incidence rate | IRR for hospital  admission rate |
| --- | --- | --- |
| 0–<12 months vs 0–<24 months | 1.13 (1.02–1.25) | 1.54 (1.45–1.64) |
| 0–<12 months vs 0–<36 months | 1.27 (1.15–1.40) | 2.08 (1.89–2.28) |
| 0–<12 months vs 0–<60 months | 1.80 (1.54–2.10) | 3.19 (2.89–3.52) |

Method of estimating RSV disease burden in 2019

As shown in **Figure S1**, we estimated the disease burden of RSV in infants for each of four outcomes, by WHO region and income level: RSV-associated ALRI incidence rates and number of episodes, hospital admission rate and number, in-hospital case fatality ratio and deaths, and overall mortality (mortality rate and number).

**Figure S1.** Approaches for estimating the baseline RSV morbidity and mortality burden in infants in 2019 by WHO region and income level.

*See the previous section for details. †Details can be found in appendix pp 16–17 of Li et al. Lancet 2022.^1^ RSV = respiratory syncytial virus. ALRI = acute lower respiratory infection. GLMM = generalised linear mixed-effects model. hCFR = in-hospital case fatality ratio.

Overall, the methods followed those in our previous publication,^1^ with an exception for the hCFR estimation for the South-East Asia Region (SEAR). Data were sparse in SEAR and a direct estimation was not possible. To address this, we leveraged the previously published hCFR estimates and hospital admission estimates by income level and estimated the hCFR in SEAR by calculating the weighted average of income-specific hCFR estimates, with the number of hospital admissions by income level within SEAR as weights. The complete formula for estimating hCFR in SEAR is shown below:

$${hCFR}^{SEAR}=\frac{\sum{hCFR}_{i}\times{hRate}_{i}\times{Pop}_{i}^{SEAR}}{\sum{hRate}_{i}\times{Pop}_{i}^{SEAR}}$$

Where $i$ denotes an income level stratum; $hCFR$, $hRate$, and $Pop$ denote in-hospital case fatality ratio, hospital admission rate and population, respectively.

Geographical distribution of data included for disease burden estimation

**Figure S2.** Location of data points included for estimating RSV-associated ALRI incidence rate.

Indirect indicates studies where incidence for the infant age group was imputed based on data from broader age groups.

**Figure S3.** Location of data points included for estimating RSV-associated ALRI hospital admission rate.

Indirect indicates studies where hospital admissions for the infant age group were imputed based on data from broader age groups.

**Figure S4.** Location of data points included for estimating RSV-associated ALRI in-hospital mortality burden.

Description of individual data points included for disease burden estimation

**Table S3.** Data points contributing to RSV-associated ALRI incidence rate estimates.

| **References** | **Country** | **Location** | **WHO region** | **Income level** | **Study period** | **Specimen** | **Diagnostic test** | **Age group** |
| --- | --- | --- | --- | --- | --- | --- | --- | --- |
| Ueno et al. 2019 | Philippines | Kawayan, Caibiran | WPR | LMIC | 2014/03-2016/06 | NPS | PCR | 0-<12 months |
| Kubale et al. 2020 | Nicaragua | Managua | AMR | LMIC | 2011-2016 | OPS | PCR | 0-<12 months |
| Hall et al. 2009 | United States | Nashville, Rochester, and Cincinnati | AMR | HIC | 2000/10-2004/09 | nasal and throat swab | PCR, culture | 0-<12 months |
| Homaira et al. 2012 | Bangladesh | Dhaka | SEAR | LIC | 2009-04/2011/03 | NPW | PCR | 0-<12 months |
| Wu et al. 2015 | Peru | San Marcos | AMR | UMIC | 2009/03-2011/09 | nasal swab | PCR | 0-<12 months |
| Krishnan et al. RSV GEN | India | Faridabad | SEAR | LMIC | 2012/08-2014/08 | OPS or NPS | PCR | 0-<12 months |
| Zar et al. RSV GEN | South Africa | Paarl | AFR | UMIC | 2012/05-2017/11 | Nasal swab | PCR | 0-<12 months |
| Stoszek et al. RSV GEN | Argentina | Rio Cuarto | AMR | UMIC | 2014/10-2017/08 | Nasal swab | PCR | 0-<12 months |
| Stoszek et al. RSV GEN | Bangladesh | Dhaka | SEAR | LMIC | 2015/08-2017/09 | Nasal swab | PCR | 0-<12 months |
| Stoszek et al. RSV GEN | Canada | Nova Scotia | AMR | HIC | 2014/01-2017/07 | Nasal swab | PCR | 0-<12 months |
| Stoszek et al. RSV GEN | Finland | Tampere, Oulu, Seinajoki and Kokkola | EUR | HIC | 2013/12-2017/09 | Nasal swab | PCR | 0-<12 months |
| Stoszek et al. RSV GEN | Honduras | San Pedro Sula | AMR | LMIC | 2014/08-2017/08 | Nasal swab | PCR | 0-<12 months |
| Stoszek et al. RSV GEN | South Africa | Johannesburg | AFR | UMIC | 2014/05-2017/08 | Nasal swab | PCR | 0-<12 months |
| Stoszek et al. RSV GEN | Thailand | Bangkok, Klongluang, and Khon Kaen | SEAR | UMIC | 2014/04-2017/07 | Nasal swab | PCR | 0-<12 months |
| Stoszek et al. RSV GEN | United States | Multiple sites | AMR | HIC | 2014/12-2017/08 | Nasal swab | PCR | 0-<12 months |
| Zar et al. RSV GEN | South Africa | Paarl | AFR | UMIC | 2012/05-2014/12 | NPS | PCR | 0-<12 months |
| Nokes et al. RSV GEN | Kenya | Kilifi | AFR | LIC | 2002/01-2005/05 | NPA, nasal wash | DFA | 0-<12 months |
| Broor et al. 2007 | India | Ballabhgarh | SEAR | LIC | 2001/10-2005/03 | NPA | DFA | 0-<12 months |
| Bruce et al. RSV GEN | Guatemala | San Marcos | AMR | LMIC | 2002/12-2004/12 | NPA | DFA | 0-<12 months |
| Brooks et al. RSV GEN | Bangladesh | Dhaka | SEAR | LIC | 2005/01-2008/02 | NPW, serum | serology, PCR | 0-<12 months |
| Simoes et al. RSV GEN | Indonesia | Bandung | SEAR | LIC | 1999/02-2001/08 | nasal wash | PCR | 0-<12 months |
| Rasmussen et al. RSV GEN | Pakistan | Gilgit | EMR | LMIC | 2012/04-2014/03 | NPS | PCR | 0-<12 months |
| Gordon et al. RSV GEN | Nicaragua | Managua | AMR | LMIC | 2014/01-2016/12 | Nasal or throat swab | PCR | 0-<12 months |
| Wright et al. RSV GEN | United States | Nashville, Tennessee | AMR | HIC | 1973/01-1993/12 | Not reported | culture | 0-<12 months |
| Forster et al. 2004 | Germany | Nationwide | EUR | HIC | 1999/11-2001/10 | NPS | PCR | 0-<36 months |
| Toivonen et al. 2020 | Finland | Southwest Findland | EUR | HIC | 2008/03-2012/02 | NPS | PCR | 0-<24 months |
| Robertson et al. 2004 | Nigeria | Ibadan | AFR | LIC | 1999/06-2001/05 | NPA, NPW | ELISA | 0-<60 months |
| Hasan et al. 2006 | Bangladesh | Mirzapur | SEAR | LIC | 1993/10-1996/08 | NPA | ELISA | 0-<24 months |
| Sutmoller et al. 1995 | Brazil | Rio de Janeiro | AMR | LMIC | 1987/01-1989/12 | NPA | IFA, culture | 0-<60 months |

RSV GEN= Respiratory Virus Global Epidemiology Network.AFR = African Region. AMR = Region of the Americas. EMR = Eastern Mediterranean Region. EUR = European Region. SEAR = South-East Asia Region. WPR = Western Pacific Region. HIC = High-income countries. UMIC = Upper-middle-income countries. LMIC = Lower-middle-income countries. LIC = Low-income countries. NPS = nasopharyngeal swab. OPS = oropharyngeal swab. NPW = nasopharyngeal wash. NPA = nasopharyngeal aspirate. PCR = polymerase chain reaction. DFA = direct fluorescent antibody assay. ELISA = enzyme-linked immunosorbent assay. IFA = immunofluorescence assay.

**Table S4.** Data points contributing to RSV-associated ALRI hospital admission rate estimates.

| **References** | **Country** | **Location** | **WHO region** | **Income level** | **Study period** | **Specimen** | **Diagnostic test** | **Age group** |
| --- | --- | --- | --- | --- | --- | --- | --- | --- |
| Moe et al. 2017 | Norway | Trondheim | EUR | HIC | 2006-2015 | NPA | PCR | 0-<12 months |
| Kubale et al. 2020 | Nicaragua | Managua | AMR | LMIC | 2011-2016 | OPS | PCR | 0-<12 months |
| Emukule et al. 2014 | Kenya | Karemo | AFR | LIC | 2009/08-2012/07 | NPS, OPS | PCR | 0-<12 months |
| Fry et al. 2010 | Thailand | Sa Kaeo and Nakhon Phanom | SEAR | LMIC | 2004/01-2007/12 | NPS, serum | PCR, IFA | 0-<12 months |
| Hall et al. 2009 | United States | Nashville, Rochester and Cincinnati | AMR | HIC | 2000/10-2004/09 | nasal and throat swab | PCR, culture | 0-<12 months |
| Iwane et al. 2004 | United States | Monroe and Davidson Counties in Tennessee | AMR | HIC | 2000/10-2001/09 | nasal and throat swab | viral culture, PCR | 0-<12 months |
| Polack et al. RSV GEN | Argentina | Buenos Aires | AMR | UMIC | 2011-2013 | NPA | PCR | 0-<12 months |
| Howie et al. RSV GEN | Gambia, The | Western Gambia | AFR | LIC | 2007/07-2008/06 | NPA | PCR | 0-<12 months |
| McCracken et al. RSV GEN | Guatemala | Santa Rosa | AMR | LMIC | 2008-2013 | NPS, OPS | PCR | 0-<12 months |
| McCracken et al. RSV GEN | Guatemala | Quetzaltenango | AMR | LMIC | 2009-2013 | NPS, OPS | PCR | 0-<12 months |
| Bassat et al. RSV GEN | Mozambique | Manhiça | AFR | LIC | 2006/09-2007/09 | NPA | PCR | 0-<12 months |
| Bassat et al. RSV GEN | Mozambique | Manhiça | AFR | LIC | 2011/01-2014/06 | NPA | PCR | 0-<12 months |
| Bont et al. RSV GEN | Netherlands | Utrecht | EUR | HIC | 2001-2010 | NPS, NPW | PCR, IF | 0-<12 months |
| Cohen et al. RSV GEN | South Africa | Gauteng province | AFR | UMIC | 2009-2012 | NPA | PCR | 0-<12 months |
| Gessner et al. RSV GEN | Togo | Tone district | AFR | LIC | 2011/08-2013/12 | nasal wash | PCR | 0-<12 months |
| Díez-Domingo et al. RSV GEN | Spain | Valencia | EUR | HIC | 2014/11-2019/07 | NPS | PCR | 0-<12 months |
| Zar et al. RSV GEN | South Africa | Paarl | AFR | UMIC | 2012/05-2017/11 | Nasal swab | PCR | 0-<12 months |
| Casalegno et al. RSV GEN | France | Lyon | EUR | HIC | 2013/10-2018/09 | Multiple | PCR or culture | 0-<12 months |
| Stoszek et al. RSV GEN | Bangladesh | Dhaka | SEAR | LMIC | 2015/08-2017/09 | Nasal swab | PCR | 0-<12 months |
| Stoszek et al. RSV GEN | Canada | Nova Scotia | AMR | HIC | 2014/01-2017/07 | Nasal swab | PCR | 0-<12 months |
| Stoszek et al. RSV GEN | Finland | Tampere, Oulu, Seinajoki and Kokkola | EUR | HIC | 2013/12-2017/09 | Nasal swab | PCR | 0-<12 months |
| Stoszek et al. RSV GEN | Honduras | San Pedro Sula | AMR | LMIC | 2014/08-2017/08 | Nasal swab | PCR | 0-<12 months |
| Stoszek et al. RSV GEN | South Africa | Johannesburg | AFR | UMIC | 2014/05-2017/08 | Nasal swab | PCR | 0-<12 months |
| Stoszek et al. RSV GEN | Thailand | Bangkok, Klongluang, and Khon Kaen | SEAR | UMIC | 2014/04-2017/07 | Nasal swab | PCR | 0-<12 months |
| Stoszek et al. RSV GEN | United States | Multiple sites | AMR | HIC | 2014/12-2017/08 | Nasal swab | PCR | 0-<12 months |
| Zar et al. RSV GEN | South Africa | Paarl | AFR | UMIC | 2012/05-2014/12 | NPS | PCR | 0-<12 months |
| Turner et al. RSV GEN | Thailand | Maela Camp, Tak Province | SEAR | LMIC | 2007/11-2010/10 | NPA | PCR | 0-<12 months |
| Yoshida et al. RSV GEN | Vietnam | Nha Trang | WPR | LMIC | 2014/01-2018/12 | NPS | PCR | 0-<12 months |
| Do et al. RSV GEN | Australia | Melbourne | WPR | HIC | 2017/08-2019/06 | Multiple | PCR | 0-<12 months |
| Noordeen et al. RSV GEN | Sri Lanka | Kegalle district | SEAR | LMIC | 2016/05-2018/07 | NPA | IFA | 0-<12 months |
| Rowlinson et al. 2017 | Egypt, Arab Rep. | Damanhour | EMR | LMIC | 2009/06-2013/12 | NPS, OPS | PCR | 0-<12 months |
| Ahmed et al. 2012 | Kenya | Dadaab and Kakuma | AFR | LIC | 2007/09-2010/08 | NPS, OPS | PCR | 0-<12 months |
| Anders et al. 2015 | Vietnam | Ho Chi Minh and Dong Thap | WPR | LMIC | 2009-2013 | NPS | PCR | 0-<12 months |
| Cilla et al. 2009 | Spain | Gipuzkoa | EUR | HIC | 2004/07-2007/06 | NPA | PCR | 0-<12 months |
| Robertson et al. 2004 | Nigeria | Ibadan | AFR | LIC | 1999/06-2001/05 | NPA, NPW | ELISA | 0-<12 months |
| Weber et al. 2002 | Gambia, The | Banjul, Fajara and Sibanor | AFR | LIC | 1993/10-1996/12 | NPA | IF | 0-<12 months |
| Nokes et al. RSV GEN | Kenya | Kilifi | AFR | LIC | 2002/01-2005/05 | NPA, nasal wash | DFA | 0-<12 months |
| Fasce et al. RSV GEN | Chile | Concepcion | AMR | HIC | 2012/01-2013/12 | NPA | IF | 0-<12 months |
| Fasce et al. RSV GEN | Chile | Iquique | AMR | HIC | 2012/01-2013/12 | NPA | IF | 0-<12 months |
| Broor et al. RSV GEN | India | Ballabhgarh | SEAR | LMIC | 2010-2012 | NPS | PCR | 0-<12 months |
| Chadha et al. RSV GEN | India | Pune | SEAR | LMIC | 2009/05-2013/04 | NPS | PCR | 0-<12 months |
| O'Brien et al. RSV GEN | United States | Navajo and WMA | AMR | HIC | 2004/10-2010/12 | NPS | PCR | 0-<12 months |
| Yoshida et al. RSV GEN | Vietnam | Nha Trang | WPR | LMIC | 2007/02-2012/12 | NPS | PCR | 0-<12 months |
| Yung et al. RSV GEN | Singapore | Singapore city | WPR | HIC | 2005/01-2015/12 | Nasopharyngeal samples | PCR or DFA | 0-<12 months |
| Ouedraogo et al. 2014 | Burkina Faso | Ouagadougou | AFR | LIC | 2010/07-2011/07 | NPA | DFA | 0-<12 months |
| Feikin et al. RSV GEN | Kenya | Bondo district | AFR | LIC | 2007/01-2009/01 | NPS, OPS | PCR | 0-<12 months |
| Jara et al. RSV GEN | Panama | David City | AMR | UMIC | 2011/01-2013/12 | NPS, OPS | IFA, PCR | 0-<12 months |
| Madhi et al. RSV GEN | South Africa | Soweto | AFR | LMIC | 1998/03-2005/10 | NPA | IF | 0-<12 months |
| Simoes et al. RSV GEN | United States | Colorado | AMR | HIC | 2008/01-2013/06 | nasal wash | PCR | 0-<12 months |
| Nokes et al. RSV GEN | Kenya | Kilifi | AFR | LIC | 2002/01-2019/12 | Multiple | Multiple | 0-<12 months |
| Soto et al. RSV GEN | Peru | Lima and Iquitos | AMR | UMIC | 2017/08-2018/07 | NPS | PCR | 0-<12 months |
| Cohen et al. RSV GEN | South Africa | Piertermaritzburg | AFR | UMIC | 2011/01-2016/12 | NPS | PCR | 0-<12 months |
| Huang et al. RSV GEN | New Zealand | Auckland | WPR | HIC | 2012/04-2015/12 | NPS or NPA | PCR | 0-<12 months |
| Cohen et al. RSV GEN | South Africa | Klerksdorp | AFR | UMIC | 2011/01-2016/12 | NPS | PCR | 0-<12 months |
| Greeberg et al. 2019 | Israel | Southern Israel | EUR | HIC | 2004/07-2012/06 | NPW | PCR, DFA | 0-<12 months |
| Chavez et al. 2019 | Bolivia | La Paz | AMR | LMIC | 2012-2017 |  | IFA | 0-<12 months |
| Chiu et al. 2010 | China | Hong Kong | WPR | HIC | 2003-2006 | NPA | DFA, culture | 0-<12 months |
| Eriksson et al. 2002 | Sweden | Stockholm | EUR | HIC | 1987-1998 | NPW | IFA | 0-<12 months |
| Oliveira et al. RSV GEN | Brazil | Belo Horizonte | AMR | UMIC | 2011-2013 | nasal swab | PCR | 0-<12 months |
| Yu et al. RSV GEN | China | Jingzhou | WPR | UMIC | 2010/01-2012/12 | NPS | PCR | 0-<12 months |
| Clara et al. RSV GEN | El Salvador | Santa Ana | AMR | LMIC | 2008-2013 | NPS | IFA | 0-<12 months |
| Campbell et al. RSV GEN | United Kingdom | Scotland | EUR | HIC | 2009/01-2016/12 | Any | PCR | 0-<12 months |
| Heikkinen et al. RSV GEN | Finland | Turku | EUR | HIC | 2006/08-2019/07 | NPS | Antigen detection or PCR | 0-<12 months |
| Moore et al. RSV GEN | Australia | Western Australia | WPR | HIC | 2000/01-2012/12 | Any | immunofluorescence, viral culture, PCR | 0-<12 months |
| Singleton et al. RSV GEN | United States | Yukon Kuskokwim, Alaska | AMR | UMIC | 2012/07-2019/06 | NPS | PCR | 0-<12 months |
| Rowlinson et al. 2013 | Egypt, Arab Rep. | Damanhour | EMR | LMIC | 2009/06-2012/06 | NPS, OPS | PCR | 0-<12 months |
| Lucero et al. RSV GEN | Philippines | Tagbilaran and Dauis | WPR | LMIC | 2000/07-2004/12 | NPA, nasal swab | culture, PCR | 0-<12 months |
| Thamthitiwat et al. RSV GEN | Thailand | Sa Kaeo and Nakhon Phanom | SEAR | UMIC | 2008/01-2011/12 | NPS | PCR | 0-<12 months |
| Singleton et al. RSV GEN | United States | Yukon Kuskokwim, Alaska | AMR | UMIC | 1994/07-2012/06 | NPS, NPW | PCR | 0-<12 months |
| Markic et al. RSV GEN | Croatia | Split | EUR | HIC | 2014/01-2019/12 | Nasal aspirate | Antigen Test | 0-<12 months |
| Greenberg et al. 2020 | Israel | Southern Israel | EUR | HIC | 2004/07-2012/06 | NPW | DFA | 0-<12 months |
| Boyce et al. 2000 | United States | Tennessee | AMR | HIC | 1989/07-1993/06 | Not reported | Not reported | 0-<12 months |
| Muller-Pebody et al. 2002 | United Kingdom | England | EUR | HIC | 1995/04-1998/03 | Not reported | Not reported | 0-<12 months |
| Svensson et al. 2015 | Sweden | Gothenburg | EUR | HIC | 2004-2011 | Not reported | PCR, IF | 0-<12 months |
| Gessner et al. RSV GEN | Indonesia | Lombok | SEAR | LIC | 2000-2002 | nasal wash | ELISA | 0-<12 months |
| O'Brien et al. RSV GEN | United States | Navajo and WMA | AMR | HIC | 1997/10-2000/03 | NPA | EIA | 0-<12 months |
| Gilca et al. 2020 | Canada | Nunavik | AMR | HIC | 2014/11-2019/06 | NPS | Rapid antigen detection tests (RADT) | 0-<12 months |
| Hamrin et al. 2020 | Sweden | Stockholm | EUR | HIC | 2008-2016 | Not reported | IFA | 0-<12 months |
| Fjaerli et al. 2004 | Norway | Lørenskog | EUR | HIC | 1993/02-2000/01 | NPA | ELISA | 0-<12 months |
| Gil-Prieto et al. 2015 | Spain | Nationwide | EUR | HIC | 1997/01-2011/12 | Not reported | Not reported | 0-<12 months |
| Holman et al. 2004 | United States | Nationwide and Alaska | AMR | HIC | 2000/01-2001/12 | Not reported | Not reported | 0-<12 months |
| Leader et al. 2003 | United States | Nationwide | AMR | HIC | 1997-2000 | Not reported | Not reported | 0-<12 months |
| Stockman et al. 2012 | United States | Nationwide | AMR | HIC | 1997-2006 | Not reported | Not reported | 0-<12 months |
| Weigl et al. 2002 | Germany | Kiel | EUR | HIC | 1996-2001 | NPA | PCR | 0-<12 months |
| Munoz-Quiles et al. 2016 | Spain | Valencia | EUR | HIC | 2009/01-2012/12 | Not reported | Not reported | 0-<12 months |
| Vicente et al. 2003 | Spain | Gipuzoka | EUR | HIC | 1996/07-2000/06 | NPA | ELISA | 0-<12 months |
| Whitehall et al. 2001 | Australia | Townsville | WPR | HIC | 1997/01-1999/10 | Not reported | IF, culture | 0-<12 months |
| Rabarison et al. 2018 | Madagascar | Antananaviro | AFR | LIC | 2011/01-2016/12 | NPS, OPS | PCR | 0-<60 months |
| Forster et al. 2004 | Germany | Nationwide | EUR | HIC | 1999/11-2001/10 | NPS | PCR | 0-<36 months |
| Homaira et al. 2012 | Bangladesh | Dhaka | SEAR | LIC | 2009-04/2011/03 | NPW | PCR | 0-<60 months |
| Jain et al. 2015 | United States | Memphis, Nashville, and Salt Lake City | AMR | HIC | 2010/07-2012/06 | NPS, OPS | PCR | 0-<24 months |
| Homaira et al. RSV GEN | Bangladesh | Kishoregonj, Bogra, Comilla, and Barisal | SEAR | LIC | 2010-2013 | nasal and throat swab | PCR | 0-<60 months |
| Kamigaki et al. 2017 | Philippines | Baguio | WPR | LMIC | 2012/01-2014/12 | NPS, OPS | PCR | 0-<60 months |
| Toivonen et al. 2020 | Finland | Southwest Finland | EUR | HIC | 2008/03-2012/02 | NPS | PCR | 0-<24 months |
| Hacimustafaoglu et al. 2013 | Turkey | Bursa | EUR | UMIC | 2010/03-2011/02 | nasal swab | IF | 0-<24 months |
| Suwanjutha et al. 2002 | Thailand | Takhli | SEAR | LMIC | 1999-2000 | NPA | IF | 0-<60 months |
| Ji et al. 2010 | China | Suzhou | WPR | LMIC | 2007/01-2008/12 | nasal aspirate | DFA | 0-<60 months |
| Resch et al. 2002 | Austria | Graz | EUR | HIC | 1999/11-2000/10 | NPA | ELISA | 0-<24 months |
| Chan et al. 1999 | China | Hong Kong | WPR | HIC | 1993/01-1997/12 | NPA, throat swab | DFA, virus isolation | 0-<60 months |
| Dede et al. 2010 | Australia | Alice Springs | WPR | HIC | 2000/01-2004/12 | NPA | DFA | 0-<24 months |
| Henrickson et al. 2004 | United States | Milwaukee | AMR | HIC | 1996/11-1998/10 | NPS, throat swab | culture, ELISA | 0-<60 months |
| Kassem et al. 2019 | Israel | Hadera sub-district | EUR | HIC | 2008/01-2011/12 | NPS, NPA | Immunochromatographic assay | 0-<24 months |
| Deshpande et al. 2003 | United Kingdom | Shropshire | EUR | HIC | 1996/04-1999/03 | Not reported | IF | 0-<24 months |
| Diez Domingo et al. 2006 | Spain | Valencia | EUR | HIC | 2001-2002 | Not reported | Not reported | 0-<24 months |
| Paramore et al. 2004 | United States | Nationwide | AMR | HIC | 2000/01-2000/12 | Not reported | Not reported | 0-<60 months |

RSV GEN= Respiratory Virus Global Epidemiology Network.AFR = African Region. AMR = Region of the Americas. EMR = Eastern Mediterranean Region. EUR = European Region. SEAR = South-East Asia Region. WPR = Western Pacific Region. HIC = High-income countries. UMIC = Upper-middle-income countries. LMIC = Lower-middle-income countries. LIC = Low-income countries. NPS = nasopharyngeal swab. OPS = oropharyngeal swab. NPW = nasopharyngeal wash. NPA = nasopharyngeal aspirate. PCR = polymerase chain reaction. IF/IFA = immunofluorescence assay. DFA = direct fluorescent antibody assay.

**Table S5.** Studies included for estimating in-hospital case fatality ratios (in-hospital CFR).

| **References** | **Country** | **Location** | **WHO region** | **Income level** | **Study period** | **Specimen** | **Diagnostic test** |
| --- | --- | --- | --- | --- | --- | --- | --- |
| Whitehall et al. 2001 | Australia | Townsville | WPR | HIC | 1997/01-1999/10 | Not reported | IF, culture |
| Eriksson et al. 2002 | Sweden | Stockholm | EUR | HIC | 1987-1998 | NPW | IFA |
| Resch et al. 2002 | Austria | Graz | EUR | HIC | 1999/11-2000/10 | NPA | ELISA |
| Tsolia et al. 2003 | Greece | Athens | EUR | HIC | 1997/02-2000/06 | NPW | DFA |
| Simon et al. 2007 | Germany | Nationwide | EUR | HIC | 1999-2005 | NPA | ELISA, culture, PCR |
| Savic et al. 2011 | Serbia | Belgrade | EUR | UMIC | 2008/11-2009/03 | NPA | EIA |
| El Kholy et al. 2013 | Egypt, Arab Rep. | Cairo | EMR | LMIC | 2010/02-2011/03 | NPS and OPS | PCR |
| Gil-Prieto et al. 2015 | Spain | Nationwide | EUR | HIC | 1997/01-2011/12 | Not reported | Not reported |
| Vizcarra et al. 2016 | Mexico | San Luis Potosi | AMR | UMIC | 2003/05-2014/12 | NPA | Not reported |
| Ng et al. 2016 | Malaysia | Seremban | WPR | UMIC | 2008/01-2013/12 | NPA | DFA |
| Ali et al. 2017 | Pakistan | Karachi | EMR | LMIC | 2009/08-2012/07 | OPS | PCR |
| Pale et al. 2017 | Mozambique | Maputo | AFR | LIC | 2015/01-2016/01 | NPS | PCR |
| Horton et al. 2017 | Egypt | Multiple location | AMR | UMIC | 2007/12-2014/02 | Not reported | PCR |
| Horton et al. 2017 | Jordan | Multiple location | EMR | UMIC | 2008/12-2014/02 | Not reported | PCR |
| Horton et al. 2017 | Oman | Multiple location | EMR | HIC | 2008/12-2013/02 | Not reported | PCR |
| Horton et al. 2017 | Qatar | Multiple location | EMR | HIC | 2010/12-2013/02 | Not reported | PCR |
| Horton et al. 2017 | Yemen | Multiple location | EMR | LMIC | 2010/12-2014/02 | Not reported | PCR |
| Ng et al. 2017 | Malaysia | Seremban | WPR | UMIC | 2008/01-2013/12 | secretions (unspecified) | DFA |
| Amini et al. 2019 | Canada | Quebec | AMR | HIC | 2012/01-2013/12 | NPS | PCR |
| Greeberg et al. 2019 | Israel | Southern Israel | EUR | HIC | 2004/07-2012/06 | NPW | PCR, DFA |
| Arias et al. 2019 | Paraguay | Asunción | AMR | UMIC | 2017/01-2018/10 | NPS | PCR, IFA |
| Kubale et al. 2020 | Nicaragua | Managua | AMR | LMIC | 2011-2016 | OPS | PCR |
| Greenberg et al. 2020 | Israel | Southern Israel | EUR | HIC | 2004/07-2012/06 | NPW | DFA |
| Resch et al. 2020 | Austria | Sourthern Austria | EUR | HIC | 2009/01-2015/12 | NPA | EIA |
| Polack et al. RSV GEN | Argentina | Buenos Aires | AMR | UMIC | 2011-2013 | NPA | PCR |
| Howie et al. RSV GEN | Gambia, The | Westernn Gambia | AFR | LIC | 2007/07-2008/06 | NPA | PCR |
| McCracken et al. RSV GEN | Guatemala | Santa Rosa | AMR | LMIC | 2008-2013 | NPS, OPS | PCR |
| McCracken et al. RSV GEN | Guatemala | Quetzaltenango | AMR | LMIC | 2009-2013 | NPS, OPS | PCR |
| Bassat et al. RSV GEN | Mozambique | Manhiça | AFR | LIC | 2006/09-2007/09 | NPA | PCR |
| Bassat et al. RSV GEN | Mozambique | Manhiça | AFR | LIC | 2011/01-2014/06 | NPA | PCR |
| Bont et al. RSV GEN | Netherlands | Utrecht | EUR | HIC | 2001-2010 | NPS, NPW | PCR, IF |
| Cohen et al. RSV GEN | South Africa | Gauteng province | AFR | UMIC | 2009-2012 | NPA | PCR |
| Gessner et al. RSV GEN | Togo | Tone district | AFR | LIC | 2011/08-2013/12 | nasal wash | PCR |
| Goyet et al. RSV GEN | Cambodia | Takeo and Kampong Cham | WPR | LIC | 2007/04-2010/02 | NPS, throat swab | PCR |
| Khuri-Bulos et al. RSV GEN | Jordan | Amman | EMR | UMIC | 2010/03-2013/03 | nasal and throat swab | PCR |
| Bassat et al. RSV GEN | Morocco | Rabat | EMR | LMIC | 2010/11-2011/12 | NPA | PCR |
| Ali et al. RSV GEN | Pakistan | Karachi | EMR | LMIC | 2009/08-2012/07 | throat swab | PCR |
| Cohen et al. RSV GEN | South Africa | Mpumalanga | AFR | UMIC | 2010/01-2014/12 | NPA | PCR |
| Díez-Domingo et al. RSV GEN | Spain | Valencia | EUR | HIC | 2014/11-2019/07 | NPS | PCR |
| Zar et al. RSV GEN | South Africa | Paarl | AFR | UMIC | 2012/05-2017/11 | Nasal swab | PCR |
| Zar et al. RSV GEN | South Africa | Paarl | AFR | UMIC | 2012/05-2014/12 | NPS | PCR |
| Gentile et al. RSV GEN | Argentina | Buenos Aires | AMR | UMIC | 2001/01-2013/12 | NPA | IFA |
| Rath et al. RSV GEN | Germany | Berlin | EUR | HIC | 2010/04-2014/03 | NPS, NPA | PCR |
| PERCH et al. RSV GEN | Gambia, The | Basse | AFR | LIC | 2011/11-2013/11 | NPS, OPS | PCR |
| PERCH et al. RSV GEN | Kenya | Kilifi | AFR | LIC | 2011/08-2013/11 | NPS, OPS | PCR |
| PERCH et al. RSV GEN | Mali | Bamako | AFR | LIC | 2012/01-2014/01 | NPS, OPS | PCR |
| PERCH et al. RSV GEN | Thailand | Sa Kaeo and Nakhom Phanom | SEAR | UMIC | 2012/01-2014/01 | NPS, OPS | PCR |
| PERCH et al. RSV GEN | Zambia | Lusaka | AFR | LMIC | 2011/10-2013/10 | NPS, OPS | PCR |
| PERCH et al. RSV GEN | Bangladesh | Dhaka | SEAR | LIC | 2012/01-2013/12 | NPS, OPS | PCR |
| PERCH et al. RSV GEN | Bangladesh | Matlab | SEAR | LIC | 2012/01-2013/12 | NPS, OPS | PCR |
| Lupisan et al. RSV GEN | Philippines | Tacloban | WPR | LMIC | 2008/05-2012/07 | NPS, serum | PCR, virus isolation, culture, serology |
| Gentile et al. RSV GEN | Argentina | Buenos Aires | AMR | HIC | 2014/01-2019/12 | NPA | IFA |
| Yoshida et al. RSV GEN | Vietnam | Nha Trang | WPR | LMIC | 2014/01-2018/12 | NPS | PCR |
| Do et al. RSV GEN | Australia | Melbourne | WPR | HIC | 2017/08-2019/06 | Multiple | PCR |
| Lopez et al. RSV GEN | Argentina | Buenos Aires | AMR | UMIC | 2018/05-2019/12 | NPA | PCR or IFA |
| Fasce et al. RSV GEN | Chile | Concepcion | AMR | HIC | 2012/01-2013/12 | NPA | IF |
| Fasce et al. RSV GEN | Chile | Iquique | AMR | HIC | 2012/01-2013/12 | NPA | IF |
| Broor et al. RSV GEN | India | Ballabhgarh | SEAR | LMIC | 2010-2012 | NPS | PCR |
| Chadha et al. RSV GEN | India | Pune | SEAR | LMIC | 2009/05-2013/04 | NPS | PCR |
| O'Brien et al. RSV GEN | United States | Navajo and WMA | AMR | HIC | 2004/10-2010/12 | NPS | PCR |
| Yoshida et al. RSV GEN | Vietnam | Nha Trang | WPR | LMIC | 2007/02-2012/12 | NPS | PCR |
| GABRIEL et al. RSV GEN | China | Beijing | WPR | UMIC | 2011/01-2012/12 | NPA, NPS | PCR |
| GABRIEL et al. RSV GEN | Mongolia | Ulaanbaatar | WPR | LMIC | 2011/09-2012/10 | NPA, NPS | PCR |
| GABRIEL et al. RSV GEN | Cambodia | Phnom Penh | WPR | LIC | 2010/10-2013/01 | NPA, NPS | PCR |
| GABRIEL et al. RSV GEN | India | Lucknow | SEAR | LMIC | 2012/06-2013/12 | NPA, NPS | PCR |
| GABRIEL et al. RSV GEN | Madagascar | Antananarivo | AFR | LIC | 2010/12-2013/02 | NPA, NPS | PCR |
| GABRIEL et al. RSV GEN | Paraguay | Asuncion | AMR | LMIC | 2010/07-2013/05 | NPA, NPS | PCR |
| GABRIEL et al. RSV GEN | Mali | Bamako | AFR | LIC | 2011/07-2012/11 | NPA, NPS | PCR |
| Montgomery et al. RSV GEN | Kenya | Lwak | AFR | LIC | 2007-2011 | NPS, OPS | PCR |
| Echavarria et al. RSV GEN | Argentina | Buenos Aires | AMR | UMIC | 2008/05-2019/10 | NPA | IFA |
| Feikin et al. RSV GEN | Kenya | Bondo district | AFR | LIC | 2007/01-2009/01 | NPS, OPS | PCR |
| Jara et al. RSV GEN | Panama | David City | AMR | UMIC | 2011/01-2013/12 | NPS, OPS | IFA, PCR |
| Madhi et al. RSV GEN | South Africa | Soweto | AFR | LMIC | 1998/03-2005/10 | NPA | IF |
| Simoes et al. RSV GEN | United States | Colorado | AMR | HIC | 2008/01-2013/06 | nasal wash | PCR |
| Nokes et al. RSV GEN | Kenya | Kilifi | AFR | LIC | 2002/01-2019/12 | Multiple | Multiple |
| Soto et al. RSV GEN | Peru | Lima and Iquitos | AMR | UMIC | 2017/08-2018/07 | NPS | PCR |
| Cohen et al. RSV GEN | South Africa | Piertermaritzburg | AFR | UMIC | 2011/01-2016/12 | NPS | PCR |
| Jullien et al. RSV GEN | Bhutan | Thimphu | SEAR | LMIC | 2017/07-2018/06 | NP washing | PCR |
| Huang et al. RSV GEN | New Zealand | Auckland | WPR | HIC | 2012/04-2015/12 | NPS or NPA | PCR |
| Bandeira et al. RSV GEN | Portugal | Lisbon | EUR | HIC | 2015/01-2019/12 | NPS or respiratory secretions | PCR or RAPD |
| Seo et al. RSV GEN | Korea, Rep. | Goyang-si | WPR | HIC | 2010/01-2019/12 | Nasal swab | PCR |
| Cohen et al. RSV GEN | South Africa | Klerksdorp | AFR | UMIC | 2011/01-2016/12 | NPS | PCR |
| Oliveira et al. RSV GEN | Brazil | Belo Horizonte | AMR | UMIC | 2011-2013 | nasal swab | PCR |
| Clara et al. RSV GEN | El Salvador | Santa Ana | AMR | LMIC | 2008-2013 | NPS | IFA |
| Campbell et al. RSV GEN | United Kingdom | Scotland | EUR | HIC | 2009/01-2016/12 | Any | PCR |
| Heikkinen et al. RSV GEN | Finland | Turku | EUR | HIC | 2006/08-2019/07 | NPS | Antigen detection or PCR |
| Moore et al. RSV GEN | Australia | Western Australia | WPR | HIC | 2000/01-2012/12 | Any | immunofluorescence, viral culture, PCR |
| Chakhunashvili et al. RSV GEN | Georgia | Tbilisi, Kutaisi | EUR | LMIC | 2015/09-2018/12 | NPS | PCR |
| Lucero et al. RSV GEN | Philippines | Tagbilaran and Dauis | WPR | LMIC | 2000/07-2004/12 | NPA, nasal swab | culture, PCR |
| Thamthitiwat et al. RSV GEN | Thailand | Sa Kaeo and Nakhon Phanom | SEAR | UMIC | 2008/01-2011/12 | NPS | PCR |
| Markic et al. RSV GEN | Croatia | Split | EUR | HIC | 2014/01-2019/12 | Nasal aspirate | Antigen Test |
| Gessner et al. RSV GEN | Indonesia | Lombok | SEAR | LIC | 2000-2002 | nasal wash | ELISA |
| O'Brien et al. RSV GEN | United States | Navajo and WMA | AMR | HIC | 1997/10-2000/03 | NPA | EIA |
| Salimi et al. RSV GEN | Iran, Islamic Rep. | Tehran | EMR | UMIC | 2015/12-2019/05 | NPS | PCR |
| Baibus et al. RSV GEN | Russian Federation | Saint Petersburg | EUR | UMIC | 2013/01-2019/05 | Not reported | Not reported |
| Baibus et al. RSV GEN | Russian Federation | 6 cities | EUR | UMIC | 2012/10-2019/09 | Not reported | Not reported |

RSV GEN= Respiratory Virus Global Epidemiology Network.AFR = African Region. AMR = Region of the Americas. EMR = Eastern Mediterranean Region. EUR = European Region. SEAR = South-East Asia Region. WPR = Western Pacific Region. HIC = High-income countries. UMIC = Upper-middle-income countries. LMIC = Lower-middle-income countries. LIC = Low-income countries. NPS = nasopharyngeal swab. OPS = oropharyngeal swab. NPA = nasopharyngeal aspirate. NPW = nasopharyngeal wash. PCR = polymerase chain reaction. IF/IFA = immunofluorescence assay. DFA = direct fluorescent antibody assay. ELISA = enzyme-linked immunosorbent assay. EIA = Enzyme Immunoassay. RAPD = randomly amplified polymorphic DNA.

List of countries with nirsevimab or RSVpreF licensure or NIP inclusion as of June 2025

**Table S6.** List of countries with nirsevimab or RSVpreF licensure or NIP inclusion as of June 2025.

| Country | nirsevimab | RSVpreF | WHO region | Income level |
| --- | --- | --- | --- | --- |
| United States | NIP* | NIP | AMR | HIC |
| Belgium | NIP* | NIP | EUR | HIC |
| France | NIP* | NIP | EUR | HIC |
| Luxembourg | NIP* | NIP | EUR | HIC |
| Switzerland | NIP* | NIP | EUR | HIC |
| Australia | NIP | NIP* | WPR | HIC |
| Canada | NIP | licensed | AMR | HIC |
| Chile | NIP | licensed | AMR | HIC |
| Austria | NIP | licensed | EUR | HIC |
| Finland | NIP | licensed | EUR | HIC |
| Germany | NIP | licensed | EUR | HIC |
| Ireland | NIP | licensed | EUR | HIC |
| Italy | NIP | licensed | EUR | HIC |
| Portugal | NIP | licensed | EUR | HIC |
| Spain | NIP | licensed | EUR | HIC |
| Paraguay | NIP | unlicensed | AMR | UMIC |
| Argentina | licensed | NIP | AMR | UMIC |
| Uruguay | licensed | NIP | AMR | HIC |
| Poland | licensed | NIP | EUR | HIC |
| Slovenia | licensed | NIP | EUR | HIC |
| United Kingdom | licensed | NIP | EUR | HIC |
| Brazil | licensed | licensed | AMR | UMIC |
| Panama | licensed | licensed | AMR | HIC |
| Bahrain | licensed | licensed | EMR | HIC |
| Kuwait | licensed | licensed | EMR | HIC |
| Saudi Arabia | licensed | licensed | EMR | HIC |
| United Arab Emirates | licensed | licensed | EMR | HIC |
| Bulgaria | licensed | licensed | EUR | UMIC |
| Croatia | licensed | licensed | EUR | HIC |
| Cyprus | licensed | licensed | EUR | HIC |
| Czechia | licensed | licensed | EUR | HIC |
| Denmark | licensed | licensed | EUR | HIC |
| Estonia | licensed | licensed | EUR | HIC |
| Greece | licensed | licensed | EUR | HIC |
| Hungary | licensed | licensed | EUR | HIC |
| Latvia | licensed | licensed | EUR | HIC |
| Lithuania | licensed | licensed | EUR | HIC |
| Malta | licensed | licensed | EUR | HIC |
| Netherlands | licensed | licensed | EUR | HIC |
| Romania | licensed | licensed | EUR | HIC |
| Slovakia | licensed | licensed | EUR | HIC |
| Sweden | licensed | licensed | EUR | HIC |
| Iceland | licensed | licensed | EUR | HIC |
| Israel | licensed | licensed | EUR | HIC |
| Norway | licensed | licensed | EUR | HIC |
| Turkey | licensed | licensed | EUR | UMIC |
| Thailand | licensed | licensed | SEAR | UMIC |
| Japan | licensed | licensed | WPR | HIC |
| Malaysia | licensed | licensed | WPR | UMIC |
| Singapore | licensed | licensed | WPR | HIC |
| Liechtenstein | licensed | licensed |  | NAIC |
| India | licensed | unlicensed | SEAR | LMIC |
| China | licensed | unlicensed | WPR | UMIC |
| Republic of Korea | licensed | unlicensed | WPR | HIC |
| Costa Rica | unlicensed | NIP | AMR | UMIC |
| Ghana | unlicensed | licensed | AFR | LMIC |
| South Africa | unlicensed | licensed | AFR | UMIC |
| Dominican Republic | unlicensed | licensed | AMR | UMIC |
| El Salvador | unlicensed | licensed | AMR | LMIC |
| Mexico | unlicensed | licensed | AMR | UMIC |
| Peru | unlicensed | licensed | AMR | UMIC |
| Egypt | unlicensed | licensed | EMR | LMIC |
| Oman | unlicensed | licensed | EMR | HIC |
| Pakistan | unlicensed | licensed | EMR | LMIC |
| Qatar | unlicensed | licensed | EMR | HIC |
| Belarus | unlicensed | licensed | EUR | UMIC |
| Indonesia | unlicensed | licensed | SEAR | UMIC |
| Philippines | unlicensed | licensed | WPR | LMIC |

NIP=National Immunization Program. *indicates the primarily used product based on WHO internal summary data. AFR = African Region. AMR = Region of the Americas. EMR = Eastern Mediterranean Region. EUR = European Region. SEAR = South-East Asia Region. WPR = Western Pacific Region. HIC = High-income countries. UMIC = Upper-middle-income countries. LMIC = Lower-middle-income countries. LIC = Low-income countries. Detailed information is available via the interactive webpage: <https://idem.njmu.edu.cn/shiny/rsvpassiveimmutracker/>.

Country-level uptake of nirsevimab and RSVPreF

For the nine countries with available real-world uptake data,^3–8^ France, Luxembourg, United States, Spain, Italy, Ireland, Chile, United Kingdom, and Argentina we used the corresponding reported uptake data (**Table S7**); for the rest countries, we assumed varied uptake levels by the implementation status of the two products (**Table S8**). Our primary assumptions (for the main analysis) were as follows:

i. When both nirsevimab and RSVpreF were included in the national immunization program (NIP), the uptake of the primary product was first determined. Subsequently, the uptake of the non-primary product was calculated as *(1 − uptake of the primary product)* multiplied by a coefficient (<1), ensuring that the total uptake of nirsevimab and RSVpreF did not exceed 1.
ii. When nirsevimab was the only or primary product included in NIP, the uptake of nirsevimab was assumed to be 0.8.
iii. When RSVpreF was the only or primary product in the NIP, the uptake of RSVpreF was assumed as $min(0.6, Tdap\_c)$, where Tdap_c represents the coverage of the Tetanus, Diphtheria, and acellular Pertussis (Tdap) vaccine among pregnant women, which has an overlapping vaccination window (in late pregnancy) with RSVpreF. The reported Tdap coverage rates were 64% in Belgium,^9^ 62% in France,^10^ 79% in Australia,^11^ 86% in Switzerland,^12^ 63% in Uruguay,^13^ and 95% in Costa Rica.^10^ For countries where Tdap_c data were unavailable (Luxembourg, Poland, and Slovenia), the uptake of RSVpreF was assumed to be 0.6.
iv. When RSVpreF was included in the NIP and nirsevimab was available only in the private market, the uptake of nirsevimab was assumed to be 0.005 for upper-middle-income countries (UMIC) and 0.0005 for low- and lower-middle-income countries (LIC/LMIC); for high-income countries (HIC), as pregnant women of early-preterm-born infants (before the gestational week of 32) would not be eligible for receiving RSVPreF, we assumed that 90% of these early-preterm-born infants would receive nirsevimab; in this study, the following data on the proportions of early-preterm-born infants were used: 1% in Poland,^14^ 0.9% in Slovenia,^14^ 1.2% in UK,^14^ and 1.7% in Uruguay.^15,16^
v. When no products were included in NIP, we assumed income-specific uptake levels for the licensed products that were available only in the private market based on country income levels, as follows: for nirsevimab, 0.01 in HIC, 0.005 in UMIC, and 0.0005 in LIC/LMIC; for RSVpreF, 0.05 in HIC, 0.01 in UMIC, and 0.005 in LIC/LMIC. It is worth noting that a relatively higher uptake was assumed for RSVpreF, reflecting the anticipated lower price compared with nirsevimab in the private market.

For the assumed uptake, we also considered more conservative (i.e., lower) uptake and more optimistic (i.e., higher) uptake for sensitivity analysis (details in pp 32-34).

**Table S7.** Uptake of nirsevimab and RSVPreF among countries with available real-world uptake data.

| Countries | Income level | Implementation status | | Main analysis | | Sensitive analysis (lower uptake) | | Sensitive analysis (higher uptake) | |
| --- | --- | --- | --- | --- | --- | --- | --- | --- | --- |
|  |  | **nirsevimab** | **RSVpreF** | **nirsevimab** | **RSVpreF** | **nirsevimab** | **RSVpreF** | **nirsevimab** | **RSVpreF** |
| France | HIC | NIP* | NIP | 0.77^3^ | min((1-0.77) × 0.6,0.62) | same to main analysis | min((1-0.77) × 0.5,0.62) | same to main analysis | min(max((1-0.77) × 0.7,0.62),1-0.77) |
| Luxembourg | HIC | NIP* | NIP | 0.84^3^ | (1-0.84) × 0.6 |  | (1-0.84) × 0.5 |  | min(1-0.84) × 0.7,1-0.84) |
| United States | HIC | NIP* | NIP | 0.51^3^ | 0.31^3^ | same to main analysis | same to main analysis | same to main analysis | same to main analysis |
| Spain | HIC | NIP | licensed | 0.90^3^ | 0.0005 |  |  |  |  |
| Italy | HIC | NIP | licensed | 0.69^4^ | 0.0005 |  |  |  |  |
| Ireland | HIC | NIP | licensed | 0.83^5^ | 0.0005 |  |  |  |  |
| Chile | HIC | NIP | licensed | 0.98 and 0.90†^6^ | 0.0005 |  |  |  |  |
| United Kingdom | HIC | licensed | NIP | 1.2%‡×0.9 | 0.55^7^ | 1.2%‡×0.6 | same to main analysis | 1.2%‡×1 | same to main analysis |
| Argentina | UMIC | licensed | NIP | 0.005 | 0.62^8^ | same to main analysis |  | same to main analysis |  |

* indicates nirsevimab as the primary NIP product; Classification is based on WHO internal summary data. †0.98 for seasonal birth dose and 0.90 for catch-up dose. ‡ indicates the proportion of preterm infants born before the 32^nd^ week of gestation. HIC = High-income countries. UMIC = Upper-middle-income countries. Parameters highlighted in red indicate deviations from the main analysis.

**Table S8.** Assumed uptake of nirsevimab and RSVPreF among countries without available real-world uptake data.

| Income  level | Implementation status | | Main analysis | | Sensitive analysis (lower uptake) | | Sensitive analysis (higher uptake) | |
| --- | --- | --- | --- | --- | --- | --- | --- | --- |
|  | **nirsevimab** | **RSVpreF** | **nirsevimab(mAb_c)** | **RSVpreF(mV_c)** | **nirsevimab(mAb_c)** | **RSVpreF(mV_c)** | **nirsevimab(mAb_c)** | **RSVpreF(mV_c)** |
| Any | NIP* | NIP | 0.8 | min((1-mAb_c)×0.6, Tdap_c) | 0.6 | min((1-mAb_c)×0.5, Tdap_c) | 0.9 | min(max((1-mAb_c)×0.7,  Tdap_c) ,1-mAb_c) |
| Any | NIP | NIP† | (1-mV_c)×0.8 | min(0.6, Tdap_c) | (1-mV_c)×0.6 | min(0.5, Tdap_c) | (1-mV_c)×0.9 | max(0.7, Tdap_c) |
| Any | NIP | licensed | 0.8 | 0.0005 | 0.6 | 0.0005 | 0.9 | 0.0005 |
| Any | NIP | unlicensed | 0.8 | 0 | 0.6 | 0 | 0.9 | 0 |
| HIC | licensed | NIP | gw32_%×0.9 | min(0.6, Tdap_c) | gw32_%×0.6 | min(0.5, Tdap_c) | gw32_%×1 | max(0.7, Tdap_c) |
| UMIC | licensed | NIP | 0.005 | min(0.6, Tdap_c) | 0.005 | min(0.5, Tdap_c) | 0.005 | max(0.7, Tdap_c) |
| LIC/LMIC | licensed | NIP | 0.0005 | min(0.6, Tdap_c) | 0.0005 | min(0.5, Tdap_c) | 0.0005 | max(0.7, Tdap_c) |
| Any | unlicensed | NIP | 0 | min(0.6, Tdap_c) | 0 | min(0.5, Tdap_c) | 0 | max(0.7, Tdap_c) |
| HIC | licensed | licensed | 0.01 | 0.05 | 0.005 | 0.01 | 0.05 | 0.1 |
| UMIC | licensed | licensed | 0.005 | 0.01 | 0.0005 | 0.005 | 0.01 | 0.05 |
| LIC/LMIC | licensed | licensed | 0.0005 | 0.005 | 0.0005 | 0.0005 | 0.005 | 0.01 |
| HIC | licensed | unlicensed | 0.01 | 0 | 0.005 | 0 | 0.05 | 0 |
| UMIC | licensed | unlicensed | 0.005 | 0 | 0.0005 | 0 | 0.01 | 0 |
| LIC/LMIC | licensed | unlicensed | 0.0005 | 0 | 0.0005 | 0 | 0.005 | 0 |
| HIC | unlicensed | licensed | 0 | 0.05 | 0 | 0.01 | 0 | 0.1 |
| UMIC | unlicensed | licensed | 0 | 0.01 | 0 | 0.005 | 0 | 0.05 |
| LIC/LMIC | unlicensed | licensed | 0 | 0.005 | 0 | 0.0005 | 0 | 0.01 |
| Any | unlicensed | unlicensed | 0 | 0 | 0 | 0 | 0 | 0 |

NIP=National Immunization Program. * indicates nirsevimab and † indicates RSVpreF as the primary NIP product; Classification is based on WHO internal summary data. Parameters highlighted in red indicate changes compared to the main analysis. HIC = High-income countries. UMIC = Upper-middle-income countries. LMIC = Lower-middle-income countries. LIC = Low-income countries. gw32_% indicates the proportion of preterm infants born before the 32^nd^ week of gestation. Tdap_c represents the coverage of the Tetanus, Diphtheria, and acellular Pertussis (Tdap) vaccine among pregnant women.

Method of estimating the proportions of RSV-ALRI hospital admissions by age in months and by birth month

As countries with clear RSV seasonality tend to adopt a seasonal immunisation strategy for nirsevimab, such as a birth dose for those born during an RSV season and a catch-up dose for those under six months at the season onset, the projection of numbers of hospitalisation averted required an explicit breakdown of RSV hospitalisation by both age in months and by birth month.

In this study, we applied the same two-step Bayesian modelling framework to our previously published analysis.^17^ As the first step, we re-ran the following Bayesian hierarchical model by income level and further by WHO region –

$\vec{Y}_{s} | \left( \vec{p}_{s}, N_{s} \right) \sim\mathrm{Multinomial} \left( N_{s}, \vec{p}_{s} \right)$	(1)

$\vec{p}_{s}| \left( \alpha, \vec{p} \right) \sim\mathrm{Dirichlet}\left( \alpha\vec{p} \right), \alpha>0, \vec{p}\in\left[ 0,1 \right]^{12}, \sum_{m=1}^{12} \vec{p}_{m}=1$	(2)

where $s$ denotes each data point (i.e., study) from the same stratum of WHO region or country income level; $\vec{p}_{s}$ denotes the proportion of RSV hospitalisations in each of the 12 months of age for each data point, which follows a Dirichlet distribution; $\vec{Y}_{s}$ and $N_{s}$ are the RSV hospitalisation counts in each month of age and totally in the first year of life; α, as the concentration parameter of the Dirichlet distribution, represents the heterogeneity among studies within the same stratum. The resulted estimates, presented in **Figure S5**, were the proportion of RSV hospitalisation by each month of age, i.e.,$\vec{p}$.

As the second step, we applied country’s seasonality data for estimating the age-specific proportion ($r_{j}^{k}$) of RSV hospitalisation for a given birth month $k$

$r_{j}^{k}=q_{j}\cdot p_{\left( k+j-2 \right) mod 12 +1}$	(3)

$\vec{p} | \vec{y} \sim\mathrm{Dirichlet}\left( \vec{y} +1 \right)$	(4)

where $q_{j}$ is the posterior estimate of the age-specific proportion of RSV hospitalisation in the first step; $\vec{p}$ denotes the proportion of RSV cases in each calendar month of a year, which is simulated from a Dirichlet-Multinomial conjugate model, with a non-informative uniform $Dirichlet(1,1,\ldots,1)$ prior. The posterior distribution of the proportions is then $Dirichlet(1 + y₁, 1 + y₂, ..., 1 + y₁₂)$, where $yᵢ$are the observed case counts from each country (detailed data source is in **Table S9**). It should be noted as the model was used to project number of averted RSV cases by countries that licensed nirsevimab or RSVPreF, only RSV seasonality data from these countries were relevant to this analysis. For any countries with no identified RSV seasonality data, we assumed that its RSV seasonality was identical to a neighbouring country with available data in the same WHO region, hemisphere and latitudinal region.

**Figure S5.** Estimated proportions of RSV hospital admissions by age (months), stratified by WHO region and income level (A) or income region (B).

AFR = African Region. AMR = Region of the Americas. EMR = Eastern Mediterranean Region. EUR = European Region. SEAR = South-East Asia Region. WPR = Western Pacific Region. HIC = High-income countries. UMIC = Upper-middle-income countries. LMIC = Lower-middle-income countries. Lines indicate the medians and shaded sections indicate 95% credible intervals.

**Table S9.** Countries with nirsevimab or RSVpreF licensure or NIP inclusion, and corresponding RSV seasonality data sources.

| Country | Category of implementation status | | WHO region-hemisphere-latitudinal group | RSV seasonality data | | |
| --- | --- | --- | --- | --- | --- | --- |
|  | nirsevimab | RSVpreF |  | Data period | 6-month epidemic period^*^ | Data sources |
| Ghana | unlicensed | licensed | AFR-N-TRO | Aug 2022 – July 2023 | Jun-Nov | Published study^18^ |
| South Africa | unlicensed | licensed | AFR-S-SUB | Jan 2024 – Dec 2024 | Feb-Jul | Surveillance^19^ |
| Mexico | unlicensed | licensed | AMR-N-SUB | Jan 2024 – Dec 2024 | Sep-Feb | Surveillance^20^ |
| Canada | NIP | licensed | AMR-N-TEM | 2023/2024 season | Oct-Mar | Surveillance^21^ |
| United States | NIP | NIP | AMR-N-TEM | 2023/2024 season | Oct-Mar | Surveillance^21^ |
| Costa Rica | unlicensed | NIP | AMR-N-TRO | Jan 2024 – Dec 2024 | Jun-Nov | Surveillance^20^ |
| Dominican Republic | unlicensed | licensed | AMR-N-TRO | Jan 2024 – Dec 2024 | Jun-Nov | Surveillance^20^ |
| El Salvador | unlicensed | licensed | AMR-N-TRO | Jan 2024 – Dec 2024 | Jun-Nov | Surveillance^20^ |
| Panama | licensed | licensed | AMR-N-TRO | Jan 2024 – Dec 2024 | Jun-Nov | Surveillance^20^ |
| Uruguay | licensed | NIP | AMR-S-SUB | Jan 2019 – Dec 2019 | Apr-Sep | Published study^22^ |
| Argentina | licensed | NIP | AMR-S-TEM | Jan 2019 – Dec 2019 | Apr-Sep | Published study^23^ |
| Chile | NIP | licensed | AMR-S-TEM | Jan 2023 – Dec 2023 | Mar-Aug | Surveillance^24^ |
| Brazil | licensed | licensed | AMR-S-TRO | Jan 2019 – Dec 2019 | Jan-Jun | Published study^25^ |
| Paraguay | NIP | unlicensed | AMR-S-TRO | / | / | Assumed to be same to Brazil |
| Peru | unlicensed | licensed | AMR-S-TRO | Jan 2024 – Dec 2024 | Apr-Sep | Surveillance^20^ |
| Bahrain | licensed | licensed | EMR-N-SUB | / | / | Assumed to be same to Qatar |
| Egypt | unlicensed | licensed | EMR-N-SUB | Jan 2012 – Dec 2012 | Oct-Mar | Published study^26^ |
| Kuwait | licensed | licensed | EMR-N-SUB | Jan 2016 – Dec 2016 | Sep-Feb | Published study^27^ |
| Pakistan | unlicensed | licensed | EMR-N-SUB | 2009 – 2012 | Jul-Dec | Published study^28^ |
| Qatar | unlicensed | licensed | EMR-N-SUB | Jan 2017 – Dec 2017 | Sep-Feb | Published study^29^ |
| Saudi Arabia | licensed | licensed | EMR-N-SUB | 2015 – 2020 | Nov-Apr | Published study^30^ |
| United Arab Emirates | licensed | licensed | EMR-N-SUB | Jan 2019 – Dec 2019 | Jul-Dec | Published study^31^ |
| Oman | unlicensed | licensed | EMR-N-TRO | Jan 2008 – Dec 2008 | Sep-Feb | Published study^32^ |
| Israel | licensed | licensed | EUR-N-SUB | 2023/2024 season | Oct-Mar | Published study^33^ |
| Austria | NIP | licensed | EUR-N-TEM | 2024/2025 season | Jan-Jun | Surveillance^34^ |
| Belarus | unlicensed | licensed | EUR-N-TEM | / | / | Assumed to be same to Poland |
| Belgium | NIP | NIP | EUR-N-TEM | 2024/2025 season | Jul-Dec | Surveillance^35^ |
| Bulgaria | licensed | licensed | EUR-N-TEM | 2023/2024 season | Nov-Apr | Published study^36^ |
| Croatia | licensed | licensed | EUR-N-TEM | 2018/2019 season | Nov-Apr | Published study^37^ |
| Cyprus | licensed | licensed | EUR-N-TEM | 2024/2025 season | Jul-Dec | Surveillance^35^ |
| Czechia | licensed | licensed | EUR-N-TEM | 2018/2019 season | Nov-Apr | Surveillance^38^ |
| Denmark | licensed | licensed | EUR-N-TEM | 2024/2025 season | Nov-Apr | Surveillance^39^ |
| Estonia | licensed | licensed | EUR-N-TEM | 2024/2025 season | Jul-Dec | Surveillance^35^ |
| Finland | NIP | licensed | EUR-N-TEM | 2017 – 2019 | Dec-May | Published study^40^ |
| France | NIP | NIP | EUR-N-TEM | 2023/2024 season | Nov-Apr | Published study^41^ |
| Germany | NIP | licensed | EUR-N-TEM | 2024/2025 season | Dec-May | Surveillance^42^ |
| Greece | licensed | licensed | EUR-N-TEM | / | / | Assumed to be same to Bulgaria |
| Hungary | licensed | licensed | EUR-N-TEM | / | / | Assumed to be same to Austria |
| Iceland | licensed | licensed | EUR-N-TEM | 2024/2025 season | Nov-Apr | Surveillance^43^ |
| Ireland | NIP | licensed | EUR-N-TEM | 2024/2025 season | Nov-Apr | Surveillance^44^ |
| Italy | NIP | licensed | EUR-N-TEM | 2023/2024 season | Nov-Apr | Published study^45^ |
| Latvia | licensed | licensed | EUR-N-TEM | / | / | Assumed to be same to Denmark |
| Liechtenstein | licensed | licensed | EUR-N-TEM | / | / | Assumed to be same to Switzerland |
| Lithuania | licensed | licensed | EUR-N-TEM | 2009/2010 season | Nov-Apr | Published study^46^ |
| Luxembourg | NIP | NIP | EUR-N-TEM | 2023/2024 season | Oct-Mar | Surveillance^47^ |
| Malta | licensed | licensed | EUR-N-TEM | 2024/2025 season | Jul-Dec | Surveillance^35^ |
| Netherlands | licensed | licensed | EUR-N-TEM | 2014 – 2019 | Nov-Apr | Surveillance^48^ |
| Norway | licensed | licensed | EUR-N-TEM | 2023/2024 season | Nov-Apr | Published study^49^ |
| Poland | licensed | NIP | EUR-N-TEM | 2018/2019 season | Dec-May | Published study^50^ |
| Portugal | NIP | licensed | EUR-N-TEM | 2015 – 2018 | Aug-Jan | Published study^51^ |
| Romania | licensed | licensed | EUR-N-TEM | 2024/2025 season | Jul-Dec | Surveillance^35^ |
| Slovakia | licensed | licensed | EUR-N-TEM | / | / | Assumed to be same to Austria |
| Slovenia | licensed | NIP | EUR-N-TEM | 2017/2018 season | Feb-Jul | Published study^52^ |
| Spain | NIP | licensed | EUR-N-TEM | 2024/2025 season | Jul-Dec | Surveillance^35^ |
| Sweden | licensed | licensed | EUR-N-TEM | 2023/2024 season | Nov-Apr | Surveillance^53^ |
| Switzerland | NIP | NIP | EUR-N-TEM | 2023/2024 season | Oct-Mar | Published study^54^ |
| Turkey | licensed | licensed | EUR-N-TEM | Aug 2009 – July 2010 | Jan-Jun | Published study^55^ |
| United Kingdom | licensed | NIP | EUR-N-TEM | 2023/2024 season | Aug-Jan | Surveillance^56^ |
| India | licensed | unlicensed | SEAR-N-SUB | 2005 – 2014 | Sep-Feb | Published study^57–62^ |
| Indonesia | unlicensed | licensed | SEAR-N-TRO | 2000 – 2001 | Feb-Jul | Published study^63^ |
| Thailand | licensed | licensed | SEAR-N-TRO | 2016 – 2019 | Jul-Dec | Published study^64^ |
| China | licensed | unlicensed | WPR-N-TEM | Jan 2019 – Dec 2019 | Nov-Apr | Published study^65^ |
| Japan | licensed | licensed | WPR-N-TEM | Jan 2018 – Dec 2018 | Jul-Dec | Published study^66^ |
| Malaysia | licensed | licensed | WPR-N-TRO | 2017 – 2021 | Dec-May | Published study^67^ |
| Philippines | unlicensed | licensed | WPR-N-TRO | 2010 – 2011 | Apr-Sep | Published study^68^ |
| Republic of Korea | licensed | unlicensed | WPR-N-TEM | 2012 – 2018 | Oct-Mar | Published study^69^ |
| Singapore | licensed | licensed | WPR-N-TRO | Jan 2015 – Dec 2015 | Apr-Sep | Published study^70^ |
| Australia | NIP | NIP | WPR-S-SUB | Jan 2023 – Dec 2023 | Mar-Aug | Surveillance^71^ |

NIP=National Immunization Program. RSV=respiratory syncytial virus. N=Northern hemisphere. S=Northern Hemisphere. TRO: latitude 0°–23.5°. SUB: latitude 23.5°–35°. TEM: latitude ≥35°. AFR = African Region. AMR = Region of the Americas. EMR = Eastern Mediterranean Region. EUR = European Region. SEAR = South-East Asia Region. WPR = Western Pacific Region. **^*^**Consecutive 6 months with the highest total RSV positive cases.

Method of determining the immunisation timing of individual countries

For nirsevimab, as countries that included nirsevimab in NIP adopted a seasonal birth-dose plus catch-up implementation approach, we assumed that all rest countries adopted the same dosing strategy — infants born during the local RSV season (defined as the six consecutive months with highest RSV cases) were eligible to receive the birth dose while infants born outside the season were eligible for the catch-up dose at the beginning of the season. The detailed timing of the seasonal immunisation programme can be found in **Figures S6–S9**.

For the RSVpreF maternal vaccine, a year-round immunisation strategy is assumed by default unless a country has explicitly recommended seasonal delivery. In five countries—Austria, Belgium, the United States, Argentina, and Uruguay—where seasonal administration has been formally recommended, we assumed that all pregnant women with an expected date of delivery in the local RSV season were eligible for vaccination. For all other countries, RSVpreF was assumed to be administered year-round.

**Figure S6.** RSV seasonality in countries of the European Region (part 1).

The red colour indicates the consecutive 6-month periods with the highest total number of reported RSV-positive cases for defining RSV season. N=Northern hemisphere. S=Northern Hemisphere. TRO: latitude 0°–23.5°. SUB: latitude 23.5°–35°. TEM: latitude ≥35°.

**Figure S7.** RSV seasonality in countries of the European Region (part 2).

The red colour indicates the consecutive 6-month periods with the highest total number of reported RSV-positive cases for defining RSV season. N=Northern hemisphere. S=Northern Hemisphere. TRO: latitude 0°–23.5°. SUB: latitude 23.5°–35°. TEM: latitude ≥35°.

**Figure S8.** RSV seasonality in the WHO Region of the Americas.

The red colour indicates the consecutive 6-month periods with the highest total number of reported RSV-positive cases for defining RSV season. N=Northern hemisphere. S=Northern Hemisphere. TRO: latitude 0°–23.5°. SUB: latitude 23.5°–35°. TEM: latitude ≥35°.

**Figure S9.** RSV seasonality in countries of the Western Pacific, South-East Asia, Eastern Mediterranean, and African regions.

The red colour indicates the consecutive 6-month periods with the highest total number of reported RSV-positive cases for defining RSV season. N=Northern hemisphere. S=Northern Hemisphere. TRO: latitude 0°–23.5°. SUB: latitude 23.5°–35°. TEM: latitude ≥35°.

Method of projecting RSV-associated hospital admissions and mortality burden

We employed a static deterministic model for the projection of annual RSV-associated ALRI hospital admission and mortality burden under the two scenarios, the no-implementation scenario and the June-2025 product use scenario.

The detailed computational steps of the model are as follows. The model parameters are detailed in **Table S10.**

**Step 1**: Calculating the number of RSV-associated ALRI hospital admissions and RSV-attributable deaths among infant for each country under the no-implementation scenario

$N_{country}={Rate}_{country} \times{Pop}_{country}$	(5)

| $N_{country}$ | the number of RSV-associated ALRI hospital admissions or RSV-attributable deaths for the country under the no-implementation scenario. |
| --- | --- |
| ${Rate}_{country}$ | the country-specific RSV-associated ALRI hospital admission rate or RSV-attributable mortality rate:   - RSV-associated ALRI hospital admission rates were approximated using WHO-region- and income-level-specific estimates, or WHO-region-specific estimates (when the WHO-region- and income-level-specific estimates were unavailable). - RSV-attributable mortality rate was derived from the meta-regression model as described in **Figure S1**. |
| ${Pop}_{country}$ | the projected infant population for the country in 2025 |

**Step 2**: Calculating the number of RSV-associated ALRI hospital admissions and RSV-attributable deaths occurring in each chronological month of age in the first year of life for infants born in different birth months, based on the proportion matrix of RSV-ALRI hospital admissions by birth month and age in month (details in appendix pp 35–36).

$N_{country,k,j}=N_{country} \times r_{country,j}^{k}$	(6)

| $k$ | the birth month for the infant, with possible values for $k$ being: Jan (1), Feb (2), Mar (3), Apr (4), May (5), Jun (6), Jul (7), Aug (8), Sep (9), Oct (10), Nov (11), Dec (12). |
| --- | --- |
| $j$ | the months in age, with potential values for $j$ being: 1, 2, 3, 4, 5, 6, 7, 8, 9, 10, 11, 12. |
| $r_{j}^{k}$ | the age-specific proportion of RSV hospitalisation for infants born in month $k, \sum_{k=Jan}^{k=Dec} \sum_{j=1}^{j=12} r_{j}^{k}=1$ |
| $N_{country,k,j}$ | the number of RSV-associated ALRI hospital admissions or RSV-attributable deaths occurring between the age of *j*-1 to <*j* months for infants born in month $k$. |

**Step 3:** Calculating the averted number of RSV-associated ALRI hospital admissions or RSV-attributable deaths for each country under the June-2025 product use scenario.

$${N\_Averted}_{country}=\sum_{k=Jan}^{Dec} \sum_{j=1}^{12} {{N_{country,k,j} \times Uptake}_{country,mAb} \times Eff}_{mAb}\times{Prot}_{mAb,k,j}+$$

$\sum_{k=Jan}^{Dec} \sum_{j=1}^{12} {{N_{country,k,j} \times Uptake}_{country,vaccine} \times Eff}_{vaccine}\times{Prot}_{vaccine,k,j}$	(7)

| ${N\_Averted}_{country}$ | the total number of RSV-associated ALRI hospital admissions or RSV-attributable deaths averted by monoclonal antibody and maternal vaccine |
| --- | --- |
| ${Uptake}_{country,mAb}$ | the uptake of RSV monoclonal antibodies, depending on real-world uptakes or the implementation status of nirsevimab and RSVpreF in the country (**Tables S5–S6**) |
| ${Uptake}_{country,vaccine}$ | the uptake of RSV maternal vaccine, depending on real-world uptakes or the implementation status of nirsevimab and RSVpreF in the country (**Tables S5–S6**) |
| ${Eff}_{mAb}$ | the effectiveness of nirsevimab against RSV-associated ALRI hospitalization or RSV-attributable death |
| ${Eff}_{vaccine}$ | the effectiveness of RSVpreF against RSV-associated ALRI hospitalization or RSV-attributable death |
| ${Prot}_{mAb,k,j}$ | protection status (1 = protected, 0 = not protected) by nirsevimab for infants born in month $k$ during ages *j*-1 and <*j* months **(Figure S10)** |
| ${Prot}_{vaccine,k,j}$ | protection status (1 = protected, 0 = not protected) by RSVpreF for infants born in month $k$ during ages *j*-1 and <*j* months **(Figure S11)** |

**Step 4**: Calculating the projected annual RSV-associated ALRI hospital admissions and RSV-attributable deaths for each country, WHO region, and income level under the June-2025 product use scenario.

$$N_{country}^{'}=N_{country}-{N\_Averted}_{country}$$

$$N_{region}^{'}=\sum_{country} N_{country}^{'}$$

$$R_{region}^{'}=\frac{N_{region}^{'}}{\sum_{country} {Pop}_{country}}$$

| $N_{country}^{'}$ | the number of RSV-associated ALRI hospital admissions or RSV-attributable deaths for each country under the June-2025 product use scenario |
| --- | --- |
| $N_{region}^{'}$ | the number of RSV-associated ALRI hospital admissions or RSV-attributable deaths for a specific WHO region or income level under the June-2025 product use scenario |
| $R_{region}^{'}$ | the projected RSV-associated ALRI hospital admission rate or RSV-attributable mortality rate for a specific WHO region or income level under the June-2025 product use scenario |

The Monte Carlo simulation method was used to evaluate the uncertainty range (UR). We generated 1,000 samples for the random-type parameters (**Table S10**) and calculated the corresponding results. The median of the results was used as the point estimate, with the 2.5th and 97.5th percentiles representing the lower and upper bounds of the uncertainty interval, respectively.

**Table S10.** Overview of model parameters and sources.

| Parameter | Value or source | Type |
| --- | --- | --- |
| *Disease burden and population* | | |
| RSV-associated ALRI hospital admission rate | Region-income specific or region-specific (be used when region-income specific estimates was unavailable) hospital admission rate estimated above | Random draws from the meta-estimation |
| RSV-attributable mortality rate | Country-specific mortality estimated above | Random draws from the meta-estimation |
| Infant population in 2025 | Data from United Nations (UN)^72^ | Fixed value |
| *Seasonality associated* parameters | | |
| Proportions of RSV-ALRI hospital admissions by age in month and by birth month | Estimated using a Bayesian model (details in pp 35–36) | Random draws from the model posteriors |
| *Uptake of RSV prophylactic product* | | |
| Uptake of mAb and maternal vaccine | Real-world uptake or assumed uptake, depending on data availability (details in pp 30-34) | Fixed value |
| *Protection effect of RSV prophylactic product* | | |
| Effectiveness of long-acting mAb | - against RSV-ALRI hospital admission: 0.83 (main analysis),  0.78 or 0.88 (sensitivity analysis)  - against RSV-attributable death: 0.81 (main analysis),  0.76 or 0.86 (sensitivity analysis) | Fixed value |
| Effectiveness of maternal vaccine | - against RSV-ALRI hospital admission: 0.71 (main analysis),  0.66 or 0.76 (sensitivity analysis)  - against RSV-attributable death: 0.77 (main analysis),  0.72 or 0.82 (sensitivity analysis) | Fixed value |
| Protect duration of mAb and maternal vaccine | Six months^73,74^ | Fixed value |

**Figure S10**. Illustration of the seasonal with catch-up administration strategy for nirsevimab.

Arrows indicate the timing of administration.

**Figure S11**. Illustration of the seasonal (A) and year-round (B) vaccination strategy for RSVpreF maternal vaccine.

Arrows indicate the timing of vaccination. *The month of delivery, rather than the month of maternal vaccination, is aligned with the timing of the local RSV season.

Supplementary results

Comparison of baseline RSV morbidity and mortality burden in infants in 2019 across WHO regions.

**Figure S12.** Comparison of baseline RSV morbidity and mortality burden in infants in 2019 across WHO regions.

RSV = respiratory syncytial virus. ALRI = acute lower respiratory infection. AFR = African Region. AMR = Region of the Americas. EMR = Eastern Mediterranean Region. EUR = European Region. SEAR = South-East Asia Region. WPR = Western Pacific Region. The error bars in panel A represent the 95% confidence interval. The area of circles in panel B represents varied case/death counts across regions.

A ShinyApp tracking the implementation status of global RSV prevention products for infants

This dashboard summarises the global implementation status of RSV prevention products for infants, with data collected up to June 2025. Available at: <https://idem.njmu.edu.cn/shiny/rsvpassiveimmutracker/>

**Figure S13.** The map page of the ShinyApp.

Results of sensitivity analyses

**Figure S14.** Averted RSV-associated ALRI hospital admissions(A) and percentage reduction (B) annually under the June-2025 product use scenario compared with no-implementation scenario.

*Sensitivity analysis allowing RSV-associated hospitalisation and mortality rates under the 2025 no-implementation scenario for low-income and lower-middle-income countries to vary randomly within ±50% of the 2019 estimates. AFR = African Region. AMR = Region of the Americas. EMR = Eastern Mediterranean Region. EUR = European Region. SEAR = South-East Asia Region. WPR = Western Pacific Region. HIC = High-income countries. UMIC = Upper-middle-income countries. LMIC = Lower-middle-income countries. LIC = Low-income countries. Points indicate medians; error bars represent the 2·5th and 97·5th percentiles.

**Figure S15.** Averted RSV-attributable deaths (A) and percentage reduction (B) annually under the June-2025 product use scenario compared with no-implementation scenario.

*Sensitivity analysis allowing RSV-associated hospitalisation and mortality rates under the 2025 no-implementation scenario for low-income and lower-middle-income countries to vary randomly within ±50% of the 2019 estimates. AFR = African Region. AMR = Region of the Americas. EMR = Eastern Mediterranean Region. EUR = European Region. SEAR = South-East Asia Region. WPR = Western Pacific Region. HIC = High-income countries. UMIC = Upper-middle-income countries. LMIC = Lower-middle-income countries. LIC = Low-income countries. Points indicate medians; error bars represent the 2·5th and 97·5th percentiles.

**Figure S16.** Projected annual RSV-attributable deaths in infants by population quintile under no-implementation scenario and June-2025 product use scenario globally (panel A) and by WHO region (panel B), based on sensitivity analysis using GBD 2023 mortality data.

Error bars represent the 95% uncertainty range. Numbers in the top-left corner of each panel indicate the ratio of RSV-attributable deaths in the fifth quintile to those in the first quintile (Q5-Q1 ratio), with higher values reflecting greater disparities in RSV mortality.

**Figure S17.** Inequality in RSV-attributable infant mortality (Q5–Q1 ratio) under the no-implementation and June 2025 product use scenarios, globally and within WHO regions: a comparison between the main analysis and sensitivity analysis using GBD 2023 mortality data.

GBD = Global Burden of Disease Study. AFR = African Region. AMR = Region of the Americas. EMR = Eastern Mediterranean Region. EUR = European Region. SEAR = South-East Asia Region. WPR = Western Pacific Region. Points indicate medians; error bars represent the 2·5th and 97·5th percentiles.

**Figure S18.** Estimated annual percentage of RSV-attributable deaths averted under the June 2025 product use scenario relative to the no-implementation scenario: a comparison between main analysis and sensitivity analysis using GBD 2023 mortality data.

AFR = African Region. AMR = Region of the Americas. EMR = Eastern Mediterranean Region. EUR = European Region. SEAR = South-East Asia Region. WPR = Western Pacific Region. HIC = High-income countries. UMIC = Upper-middle-income countries. LMIC = Lower-middle-income countries. LIC = Low-income countries. Points indicate medians; error bars represent the 2·5th and 97·5th percentiles.

**Figure S19. Sensitivity analyses of changes in the Q5–Q1 ratio for RSV-attributable infant mortality, globally and by WHO region.**

*Sensitivity analysis allowing RSV-associated hospitalisation and mortality rates under the 2025 no-implementation scenario for low-income and lower-middle-income countries to vary randomly within ±50% of the 2019 estimates. Points indicate medians; error bars represent the 2·5th and 97·5th percentiles.

References

1	Li Y, Wang X, Blau DM, *et al.* Global, regional, and national disease burden estimates of acute lower respiratory infections due to respiratory syncytial virus in children younger than 5 years in 2019: a systematic analysis. *Lancet* 2022; **399**: 2047–64.

2	Wang X, Li Y, O’Brien KL, *et al.* Global burden of respiratory infections associated with seasonal influenza in children under 5 years in 2018: a systematic review and modelling study. *Lancet Glob Health* 2020; **8**: e497–510.

3	Trusinska D, Lee B, Ferdous S, *et al.* Real-world uptake of nirsevimab, RSV maternal vaccine, and RSV vaccines for older adults: a systematic review and meta-analysis. *eClinicalMedicine* 2025; **84**. DOI:10.1016/j.eclinm.2025.103281.

4	Consolati A, Farinelli M, Serravalle P, *et al.* Safety and Efficacy of Nirsevimab in a Universal Prevention Program of Respiratory Syncytial Virus Bronchiolitis in Newborns and Infants in the First Year of Life in the Valle d’Aosta Region, Italy, in the 2023-2024 Epidemic Season. *Vaccines (Basel)* 2024; **12**: 549.

5	National Immunisation Advisory Committee (NIAC). Updated Recommendations for the Passive Immunisation of Infants against Respiratory Syncytial Virus (RSV). Royal College of Physicians of Ireland, National Immunisation Advisory Committee (NIAC), 2025.

6	NirseCL-Monitoring the impact of nirsevimab in Chile. https://nirse.isci.cl/strategy/ (accessed Aug 5, 2025).

7	RSV maternal vaccination coverage in England: March 2025. GOV.UK. https://www.gov.uk/government/publications/rsv-immunisation-for-older-adults-and-pregnant-women-vaccine-coverage-in-england/rsv-maternal-vaccination-coverage-in-england-march-2025 (accessed Sept 26, 2025).

8	Impact and Effectiveness of Universal Respiratory Syncytial Virus Vaccination During Pregnancy on Infant Hospitalizations in Buenos Aires: A Retrospective Cohort Study. https://verixiv.org/articles/2-34 (accessed Sept 26, 2025).

9	European Centre for Disease Prevention and Control. Increase of pertussis cases in the EU/EEA: 8 May 2024. LU: Publications Office, 2024 https://data.europa.eu/doi/10.2900/831122 (accessed Oct 29, 2025).

10	Protection at birth (PAB) against neonatal tetanus vaccination coverage and vaccination coverage among pregnant women of Tetanus toxoid-containing vaccine (TT2+/Td2+) and Pertussis-containing vaccine. Immunization Data. https://immunizationdata.who.int/global/wiise-detail-page (accessed Oct 29, 2025).

11	Homaira N, He W-Q, McRae J, Macartney K, Liu B. Coverage and predictors of influenza and pertussis vaccination during pregnancy: a whole of population-based study. *Vaccine* 2023; **41**: 6522–9.

12	Lumbreras Areta M, Valiton A, Diana A, *et al.* Flu and pertussis vaccination during pregnancy in Geneva during the COVID-19 pandemic: A multicentric, prospective, survey-based study. *Vaccine* 2022; **40**: 3455–60.

13	Gentile A, Castellano VE, Ulloa-Gutierrez R, *et al.* Pertussis Vaccination During Pregnancy: Regional Situation and Impact of Implementation on National Immunization Programs in Latin America. *The Pediatric Infectious Disease Journal* 2025; **44**: S80.

14	Euro-Peristat Network. European Perinatal Health Report: Core indicators of the health and care of pregnant women and babies in Europe from 2015–2019. Paris, France: Euro-Peristat, 2022 https://www.europeristat.com/wp-content/uploads/2022/11/Euro-Peristat_Fact_sheets_2022_for_upload.pdf.

15	Chawanpaiboon S, Vogel JP, Moller A-B, *et al.* Global, regional, and national estimates of levels of preterm birth in 2014: a systematic review and modelling analysis. *The Lancet Global Health* 2019; **7**: e37–46.

16	Births, preterm, rate per 100 live births. https://www.who.int/data/gho/data/indicators/indicator-details/GHO/preterm-birth-rate-per-100-live-births (accessed Nov 2, 2025).

17	Guo L, Kenmoe S, Miyake F, *et al.* Respiratory syncytial virus hospitalisation by chronological month of age and by birth month in infants. *Nat Commun* 2025; **16**: 6109.

18	Kafintu-Kwashie AA, Nii-Trebi NI, Obodai E, Neizer M, Adiku TK, Odoom JK. Molecular epidemiological surveillance of viral agents of acute lower respiratory tract infections in children in Accra, Ghana. *BMC Pediatrics* 2022; **22**: 364.

19	National Institute for Communicable Diseases, Centre for Respiratory Diseases and Meningitis. RSV Alert for Healthcare Workers: Alert to Clinicians - Start of the Respiratory Syncytial Virus (RSV) Season. National Institute for Communicable Diseases, Division of the National Health Laboratory Service, 2025 https://www.nicd.ac.za/diseases-a-z-index/disease-index-covid-19/surveillance-reports/weekly-respiratory-pathogens-surveillance-report-week/.

20	Respiratory Syncytial Virus (RSV) Situation - Region of the Americas - PAHO/WHO | Pan American Health Organization. https://www.paho.org/en/respiratory-syncytial-virus-rsv-situation-region-americas (accessed Sept 26, 2025).

21	Respiratory Virus Hospitalization Surveillance Network (RESP-NET) | RESP-NET | CDC. https://www.cdc.gov/resp-net/dashboard/index.html (accessed Feb 19, 2025).

22	Ciapponi A, Palermo MC, Sandoval MM, *et al.* Respiratory syncytial virus disease burden in children and adults from Latin America: a systematic review and meta-analysis. *Front Public Health* 2024; **12**: 1377968.

23	Dolores A, Stephanie G, Mercedes S NJ, Érica G, Mistchenko AS, Mariana V. RSV reemergence in Argentina since the SARS-CoV-2 pandemic. *J Clin Virol* 2022; **149**: 105126.

24	Instituto de Salud Pública de Chile. https://www.ispch.gob.cl/virusrespiratorios/ (accessed Feb 19, 2025).

25	Santos RWF, Pimentel AS, de Jesus MCS, *et al.* Prevalence of respiratory viruses in children in Northeast Brazil: The scenario before the COVID-19 pandemic. *IJID Regions* 2025; **14**: 100499.

26	Rowlinson E, Dueger E, Mansour A, *et al.* Incidence and etiology of hospitalized acute respiratory infections in the Egyptian Delta. DOI:10.1111/irv.12409.

27	Madi N, Chehadeh W, Asadzadeh M, Al-Turab M, Al-Adwani A. Analysis of genetic variability of respiratory syncytial virus groups A and B in Kuwait. *Arch Virol* 2018; **163**: 2405–13.

28	Ali A, Yousafzai MT, Waris R, *et al.* RSV associated hospitalizations in children in Karachi, Pakistan: Implications for vaccine prevention strategies. *J Med Virol* 2017; **89**: 1151–7.

29	Al-Romaihi HE, Smatti MK, Ganesan N, *et al.* Epidemiology of respiratory infections among adults in Qatar (2012-2017). *PLoS One* 2019; **14**: e0218097.

30	Dhayhi NS, Aqeel A, Ghazwani S, *et al.* Five Years’ Experience with Respiratory Syncytial Virus Among Hospitalized Patients: A Retrospective Study from Jazan, Saudi Arabia. *Infect Drug Resist* 2024; **17**: 5179–87.

31	Salim S, Celiloglu H, Tayyab F, Malik ZA. Seasonal Prevalence of Respiratory Pathogens Among Children in the United Arab Emirates: A Multicenter Cross-Sectional Study in the Pre-COVID-19 Era. *Cureus* 2023; **15**: e45204.

32	Khamis FA, Al‐Kobaisi MF, Al‐Areimi WS, Al‐Kindi H, Al‐Zakwani I. Epidemiology of respiratory virus infections among infants and young children admitted to hospital in Oman. *J Med Virol* 2012; **84**: 1323–9.

33	Glatman-Freedman A, Gur-Arie L, Dichtiar R, Keinan-Boker L, Bromberg M, with the Israeli Respiratory Viruses Hospital Laboratory Network (IRVHLN). Laboratory-confirmed respiratory syncytial virus (RSV) hospitalizations: a national all ages cross-section evaluation, 2020–2024. *Israel Journal of Health Policy Research* 2025; **14**: 36.

34	SARI-Dashboard. https://www.sari-dashboard.at/ (accessed Aug 6, 2025).

35	European Respiratory Virus Surveillance Summary (ERVISS). https://erviss.org/ (accessed Feb 19, 2025).

36	Korsun N, Trifonova I, Pavlova D, *et al.* Etiological Spectrum of Acute Respiratory Infections in Bulgaria During the 2023–2024 Season and Genetic Diversity of Circulating Influenza Viruses. *Viruses* 2025; **17**: 270.

37	Mrcela D, Markic J, Zhao C, *et al.* Changes following the Onset of the COVID-19 Pandemic in the Burden of Hospitalization for Respiratory Syncytial Virus Acute Lower Respiratory Infection in Children under Two Years: A Retrospective Study from Croatia. *Viruses* 2022; **14**: 2746.

38	Jirincova H. RSV surveillance in covid times. https://archiv.szu.cz/uploads/documents/CeM/NRLs/chripka/HERA_prezentace/RSV_surveillance_in_covid_times_.pdf.

39	name. Integrated surveillance of respiratory infections in 2023/24 - combined report. https://en.ssi.dk/surveillance-and-preparedness/surveillance-in-denmark/annual-reports-on-disease-incidence/i/integrated-surveillance--of-respiratory-infections-in-2023-24---combined-report (accessed Aug 6, 2025).

40	Harding ER, Wildenbeest JG, Heikkinen T, *et al.* Inconsistent Increase in Age at Respiratory Syncytial Virus Hospitalization of Children Aged <2 Years During the Severe Acute Respiratory Syndrome Coronavirus 2 Pandemic: A Retrospective Multicenter Study in 4 European Countries. *J Infect Dis* 2024; **230**: e985–95.

41	Pinquier D, Crépey P, Tissières P, *et al.* Preventing Respiratory Syncytial Virus in Children in France: A Narrative Review of the Importance of a Reinforced Partnership Between Parents, Healthcare Professionals, and Public Health Authorities. *Infect Dis Ther* 2023; **12**: 317–32.

42	Gesundheit B für. RSV incidence rate over time. https://infektionsradar.gesund.bund.de/en/rsv/incidence (accessed Aug 8, 2025).

43	Respiratory tract infections | Ísland.is. https://island.is/en/respiratory-tract-infections (accessed Aug 6, 2025).

44	Respiratory Virus Notification Data Hub. https://respiratoryvirus.hpsc.ie/ (accessed Aug 8, 2025).

45	Lastrucci V, Pacifici M, Puglia M, *et al.* Recent trends in hospitalizations for respiratory syncytial virus after the COVID-19 pandemic and before routine immunization: Seasonality and severity updates from the 2023/2024 season in Tuscany, Italy. *International Journal of Infectious Diseases* 2025; **154**. DOI:10.1016/j.ijid.2025.107879.

46	Drazdienė N, Laan M, Zaikauskienė J, Usonis V. Respiratory syncytial virus infection in a selected sample of infants hospitalized for lower respiratory tract infection in Lithuania and Estonia. *Acta Med Litu* 2017; **24**: 191–8.

47	Respiratory Viruses in Luxembourg (ReViLux) Sentinel Network Report -Week 05. https://lns.lu/wp-content/uploads/2025/02/revilux_2025-02-05.pdf (accessed Aug 6, 2025).

48	Annual reporting on surveillance of acute respiratory infections in the Netherlands, 2023/2024 – RSV. https://www.rivm.nl/en/rsv/annual-reporting-2023-2024 (accessed Aug 6, 2025).

49	Bøås H, Seppälä E, Veneti L, *et al.* Changed epidemiology of influenza and RSV hospitalizations after the emergence of SARS-CoV-2 in Norway, 2017–2024. *Annals of Epidemiology* 2025; **108**: 77–84.

50	Mazela J, Jackowska T, Czech M, *et al.* Epidemiology of Respiratory Syncytial Virus Hospitalizations in Poland: An Analysis from 2015 to 2023 Covering the Entire Polish Population of Children Aged under Five Years. *Viruses* 2024; **16**: 704.

51	Bandeira T, Carmo M, Lopes H, *et al.* Burden and severity of children’s hospitalizations by respiratory syncytial virus in Portugal, 2015-2018. *Influenza Other Respir Viruses* 2023; **17**: e13066.

52	Grilc E, Prosenc Trilar K, Lajovic J, Sočan M. Determining the seasonality of respiratory syncytial virus in Slovenia. *Influenza Other Respir Viruses* 2021; **15**: 56–63.

53	RS-virus – sjukdomsstatistik. https://www.folkhalsomyndigheten.se/folkhalsorapportering-statistik/statistik-a-o/sjukdomsstatistik/rs-virus/ (accessed Aug 7, 2025).

54	Sauteur PMM, Plebani M, Trück J, *et al.* Ongoing disruption of RSV epidemiology in children in Switzerland. *The Lancet Regional Health – Europe* 2024; **45**. DOI:10.1016/j.lanepe.2024.101050.

55	Familyyok givenYOK. The seasonal variations of respiratory syncytial virus infections in Turkey: a 2-year epidemiological study. *The Turkish Journal of Pediatrics* 2012; **54**: 216–22.

56	Surveillance of influenza and other seasonal respiratory viruses in the UK, winter 2023 to 2024. GOV.UK. https://www.gov.uk/government/statistics/surveillance-of-influenza-and-other-seasonal-respiratory-viruses-in-the-uk-winter-2023-to-2024/surveillance-of-influenza-and-other-seasonal-respiratory-viruses-in-the-uk-winter-2023-to-2024 (accessed Aug 6, 2025).

57	Choudhary ML, Anand SP, Wadhwa BS, Chadha MS. Genetic variability of human respiratory syncytial virus in Pune, Western India. *Infect Genet Evol* 2013; **20**: 369–77.

58	Jain B, Singh AK, Dangi T, *et al.* High prevalence of human metapneumovirus subtype B in cases presenting as severe acute respiratory illness: an experience at tertiary care hospital. *Clin Respir J* 2014; **8**: 225–33.

59	Agrawal AS, Sarkar M, Ghosh S, *et al.* Prevalence of respiratory syncytial virus group B genotype BA-IV strains among children with acute respiratory tract infection in Kolkata, Eastern India. *J Clin Virol* 2009; **45**: 358–61.

60	Koul PA, Saha S, Kaul KA, *et al.* Respiratory syncytial virus among children hospitalized with severe acute respiratory infection in Kashmir, a temperate region in northern India. *J Glob Health* 2022; **12**: 04050.

61	Bharaj P, Sullender WM, Kabra SK, *et al.* Respiratory viral infections detected by multiplex PCR among pediatric patients with lower respiratory tract infections seen at an urban hospital in Delhi from 2005 to 2007. *Virol J* 2009; **6**: 89.

62	Swamy MA, Malhotra B, Reddy PVJ, Tiwari JK, Kumar N, Gupta ML. Trends of respiratory syncytial virus sub-types in children hospitalised at a tertiary care centre in Jaipur during 2012-2014. *Indian J Med Microbiol* 2017; **35**: 134–6.

63	Robertson SE, Roca A, Alonso P, *et al.* Respiratory syncytial virus infection: denominator-based studies in Indonesia, Mozambique, Nigeria and South Africa. *Bull World Health Organ* 2004; **82**: 914–22.

64	Chaiut W, Sapbamrer R, Dacha S, *et al.* Characteristics of Respiratory Syncytial Virus Infection in Hospitalized Children Before and During the COVID-19 Pandemic in Thailand. *J Prev Med Public Health* 2023; **56**: 212–20.

65	Han X, Xu P, Wang H, Mao J, Ye Q. Incident changes in the prevalence of respiratory virus among children during COVID-19 pandemic in Hangzhou, China. *J Infect* 2022; **84**: 579–613.

66	Kobayashi Y, Togo K, Agosti Y, McLaughlin JM. Epidemiology of respiratory syncytial virus in Japan: A nationwide claims database analysis. *Pediatr Int* 2022; **64**: e14957.

67	Chan CM, Wahab AA, Ali A. Determining the Relationship of Meteorological Factors and Severe Pediatric Respiratory Syncytial Virus (RSV) Infection in Central Peninsular Malaysia. *Int J Environ Res Public Health* 2023; **20**: 1848.

68	Kamigaki T, Chaw L, Tan AG, *et al.* Seasonality of Influenza and Respiratory Syncytial Viruses and the Effect of Climate Factors in Subtropical–Tropical Asia Using Influenza-Like Illness Surveillance Data, 2010 –2012. *PLoS One* 2016; **11**: e0167712.

69	Gy K, I R, Yh J, Jk K. Investigation of occurrence patterns of respiratory syncytial virus A and B in infected-patients from Cheonan, Korea. *Respiratory research* 2020; **21**. DOI:10.1186/s12931-020-01456-3.

70	Tam CC, Yeo KT, Tee N, *et al.* Burden and Cost of Hospitalization for Respiratory Syncytial Virus in Young Children, Singapore. *Emerg Infect Dis* 2020; **26**: 1489–96.

71	Respiratory Syncytial Virus (RSV) Statistics. Immunisation Coalition. https://www.immunisationcoalition.org.au/news-data/respiratory-syncytial-virus-rsv-statistics/ (accessed Feb 19, 2025).

72	World Population Prospects - Population Division - United Nations. 2024; published online Nov 21. https://population.un.org/wpp/Download/Standard/Population/ (accessed Nov 21, 2024).

73	Munro APS, Drysdale SB, Cathie K, *et al.* 180-day efficacy of nirsevimab against hospitalisation for respiratory syncytial virus lower respiratory tract infections in infants (HARMONIE): a randomised, controlled, phase 3b trial. *The Lancet Child & Adolescent Health* 2025; **9**: 404–12.

74	Kampmann B, Madhi SA, Munjal I, *et al.* Bivalent Prefusion F Vaccine in Pregnancy to Prevent RSV Illness in Infants. *N Engl J Med* 2023; **388**: 1451–64.
